# Integration of human microbiota (SIHUMIx) and zebrafish models reveals microbiome-mediated host responses to azoxystrobin

**DOI:** 10.1093/toxsci/kfag022

**Published:** 2026-03-07

**Authors:** Chloe Wray, Victor Castañeda-Monsalve, Beatrice Engelmann, Ulrike E Rolle-Kampczyk, Nicole Schweiger, Sebastian Gutsfeld, Debjyoti Ghosh, Siraz Kader, Charles R Tyler, Nico Jehmlich, Tamara Tal

**Affiliations:** Department of Ecotoxicology, Chemicals in the Environment Research Section, Helmholtz-Centre for Environmental Research—UFZ, 04318 Leipzig, Germany; Biosciences, University of Exeter, Exeter, Devon EX4 4QD, United Kingdom; Department of Molecular Toxicology, Chemicals in the Environment Research Section, Helmholtz-Centre for Environmental Research—UFZ, 04318 Leipzig, Germany; Department of Molecular Toxicology, Chemicals in the Environment Research Section, Helmholtz-Centre for Environmental Research—UFZ, 04318 Leipzig, Germany; Department of Molecular Toxicology, Chemicals in the Environment Research Section, Helmholtz-Centre for Environmental Research—UFZ, 04318 Leipzig, Germany; Department of Ecotoxicology, Chemicals in the Environment Research Section, Helmholtz-Centre for Environmental Research—UFZ, 04318 Leipzig, Germany; Department of Ecotoxicology, Chemicals in the Environment Research Section, Helmholtz-Centre for Environmental Research—UFZ, 04318 Leipzig, Germany; Department of Molecular Toxicology, Chemicals in the Environment Research Section, Helmholtz-Centre for Environmental Research—UFZ, 04318 Leipzig, Germany; Department of Ecotoxicology, Chemicals in the Environment Research Section, Helmholtz-Centre for Environmental Research—UFZ, 04318 Leipzig, Germany; Biosciences, University of Exeter, Exeter, Devon EX4 4QD, United Kingdom; Department of Molecular Toxicology, Chemicals in the Environment Research Section, Helmholtz-Centre for Environmental Research—UFZ, 04318 Leipzig, Germany; Department of Ecotoxicology, Chemicals in the Environment Research Section, Helmholtz-Centre for Environmental Research—UFZ, 04318 Leipzig, Germany; Medical Faculty, University Leipzig, 04103 Leipzig, Germany

**Keywords:** microbiome, azoxystrobin, SIHUMIx, zebrafish, metabolomics

## Abstract

The gut microbiome is essential for neurodevelopment via bidirectional gut–brain axis signaling, yet environmental chemicals can potentially disrupt this communication by altering community structure and xenobiotic metabolism. In this study, we investigated whether the fungicide azoxystrobin, a known metabolic disruptor, modulates microbiome composition and function to influence neurobehavior. We utilized a simplified human gut microbiota model (SIHUMIx) and a vertebrate host model (larval zebrafish) to elucidate microbiome-mediated mechanisms of xenobiotic neurotoxicity. SIHUMIx was exposed to azoxystrobin for 7 days at 10% of the acceptable daily intake, followed by recovery. Integrated metaproteomic and metabolomic analyses revealed functional reprogramming of the microbiota, characterized by upregulation of vitamin and cofactor biosynthesis, nutrient acquisition, and detoxification pathways, and decreases in carbohydrate fermentation and amino acid turnover, consistent with reduced short-chain fatty acid levels. Microbiome-depleted and SIHUMIx-inoculated larvae were exposed to azoxystrobin at 4 days post fertilization, and neurobehavioral outcomes were assessed after 24 h using the Visual and Acoustic Motor Response assay. Azoxystrobin exposure disrupted non-associative habituation learning independent of microbiome status but induced dark-phase hyperactivity only in colonized larvae, indicating a microbiome-dependent phenotype. Targeted metabolomics revealed lower serotonin levels in microbiome-depleted larvae relative to colonized controls and that azoxystrobin exposure reduced serotonin in colonized larvae toward depleted levels. These results suggest that microbiota-dependent serotonergic signaling may modulate host responses to azoxystrobin. This integrated ex vivo–in vivo approach supports the concept that the microbiome is a key determinant of neurotoxic responses and underscores the importance of incorporating microbiome-mediated effects into chemical risk assessment frameworks.

Azoxystrobin is a widely used (Wang et al. 2021; Battaglin et al. 2011) broad-spectrum strobilurin fungicide that inhibits mitochondrial respiration in fungi by blocking electron transport in the mitochondrial respiratory chain, ultimately preventing ATP production and inducing oxidative damage (Kim et al. 2007; Zafar et al. 2012; Du et al. 2019). Widespread application has led to its detection in surface waters, sediments, and soils, raising concerns about environmental persistence, bioaccumulation, and off-target effects (Zafar et al. 2012; Herrero-Hernández et al. 2015; Silva et al. 2019). In addition to its intended fungicidal action, azoxystrobin has been associated with adverse effects in non-target species, including the induction of oxidative stress and developmental and reproductive toxicity (Zafar et al. 2012; Cao et al. 2016; Jia et al. 2018). However, the precise mechanisms underlying these effects remain poorly understood, highlighting the need for further investigation into the pathways through which azoxystrobin exposure disrupts biological systems in non-target organisms.

One potential target of azoxystrobin is the gut microbiota, a critical modulator of xenobiotic metabolism and host health. The gut microbiota has the capacity to biotransform xenobiotics (Sousa et al. 2008; Spanogiannopoulos et al. 2016; Koppel et al. 2017), and both the parent compound and biotransformation products can potentially alter community structure (Culp et al. 2024) and function (Maurice et al. 2013; Koppel et al. 2017; Zimmermann et al. 2019). Although there are some examples showing that microbiota-dependent toxicokinetic interactions affect host health (Wallace et al. 2010, 2015; Haiser et al. 2013; Maini Rekdal et al. 2019), for most xenobiotic agents, both the molecular mechanisms and microbiome-dependent causal effects on the host are not defined. One barrier to understanding the underlying mechanisms is the lack of information on microbiome-specific chemical effects.

The simplified human gut microbiota model (SIHUMIx) provides a controlled ex vivo system for investigating chemical–microbiota interactions. Composed of 8 genetically and proteomically defined human gut isolates, SIHUMIx captures approximately 90% of the biochemical functionality of a healthy human gut microbiome (Schäpe et al. 2019). It can provide a viable cultivation for up to 21 days, with high reproducibility and well-established protocols for multiomics analyses (Schäpe et al. 2020; Petruschke et al. 2021). A previous high-throughput screening study of 90 xenobiotics in the SIHUMIx model identified azoxystrobin as a potent disruptor of microbial metabolism, with 24 h exposure significantly reducing concentrations of primary short-chain fatty acids (SCFAs) and altering pathways involved in bacterial secretion and xenobiotic metabolism (Castañeda-Monsalve et al. 2024).

Although SIHUMIx offers precise control over microbial variables, it does not account for host-mediated processes, making it difficult to determine the potential functional relevance of disrupted chemical–microbiome interactions at an organismal level. To address this limitation, we used early life-stage zebrafish (*Danio rerio*) as a complementary in vivo model. Although zebrafish and mammalian microbiota differ taxonomically (Roeselers et al. 2011; Kostic et al. 2013), they share many conserved biochemical pathways and enzymes (Bates et al. 2006; Semova et al. 2012). Moreover, established methods allow for deriving axenic (i.e., microbe-free) zebrafish (Pham et al. 2008; Milligan-McClellan et al. 2011; Melancon et al. 2017), which can be colonized via immersion with defined microbial communities at specific developmental time points (Bates et al. 2006; Weitekamp et al. 2021), including human-associated microbes (Pham et al. 2008; Toh et al. 2013; Valenzuela et al. 2018). This system enables manipulation of microbial status in the context of chemical exposure, allowing for the determination of the microbiome’s role in modulating xenobiotic effects. Behavioral assessments of early life-stage zebrafish can serve as a functional readout of neurodevelopment (Kokel et al. 2010), which is tightly regulated and influenced by host–microbiome interactions (Heijtz et al. 2011; Phelps et al. 2017). Overall, this system can reveal whether chemical-dependent effects on behavior are microbiome-dependent.

In this study, we utilized the SIHUMIx and zebrafish multi-colonization status models to determine whether azoxystrobin-induced effects are mediated by the gut microbiota. To investigate the long-term consequences of azoxystrobin–microbiota interactions using the SIHUMIx model, we extended chemical treatment to 7 days, followed by a 4-day recovery period. We applied metaproteomics to examine alterations in species abundance and metabolic pathway activities. Complementary metabolomic analyses were conducted to quantify SCFAs and capture untargeted metabolite profiles across the different cultivation stages. This approach allows for temporal resolution of structural and functional disruptions, as well as an evaluation of microbiome resilience following chemical removal. To examine potential host effects, we exposed microbiome-depleted and SIHUMIx-inoculated zebrafish larvae to azoxystrobin during neurodevelopment. We assessed behavioral outcomes, microbial composition (via 16S rRNA sequencing), and metabolite levels (including SCFAs, amino acids, and biogenic amines) to determine whether host-level effects of azoxystrobin are dependent on microbial status. By integrating a controlled microbiota system with a vertebrate host model, this work provides mechanistic insight into microbiome-mediated xenobiotic effects and clarifies potential risks posed by azoxystrobin exposure.

## Materials and methods

### Simplified human intestinal microbiota model

We employed the extended simplified human intestinal microbiota (SIHUMIx) model (Becker et al. 2011), a defined microbial consortium representing the functional core of the human gut microbiota. This community comprises 8 well-characterized human gut isolates: *Anaerostipes caccae* (DSMZ 14662), *Bacteroides thetaiotaomicron* (DSMZ 2079), *Bifidobacterium longum* (NCC 2705), *Blautia producta* (DSMZ 2950), *Clostridium butyricum* (DSMZ 10702), *Thomasclavelia ramosa* (DSMZ 1402), *Escherichia coli* K-12 (MG1655), and *Lactiplantibacillus plantarum* (DSMZ 20174). Supplementary Methods outline relevant considerations for its use (Section S2.1).

### In vitro bioreactor cultivation

**Fig. 1.**
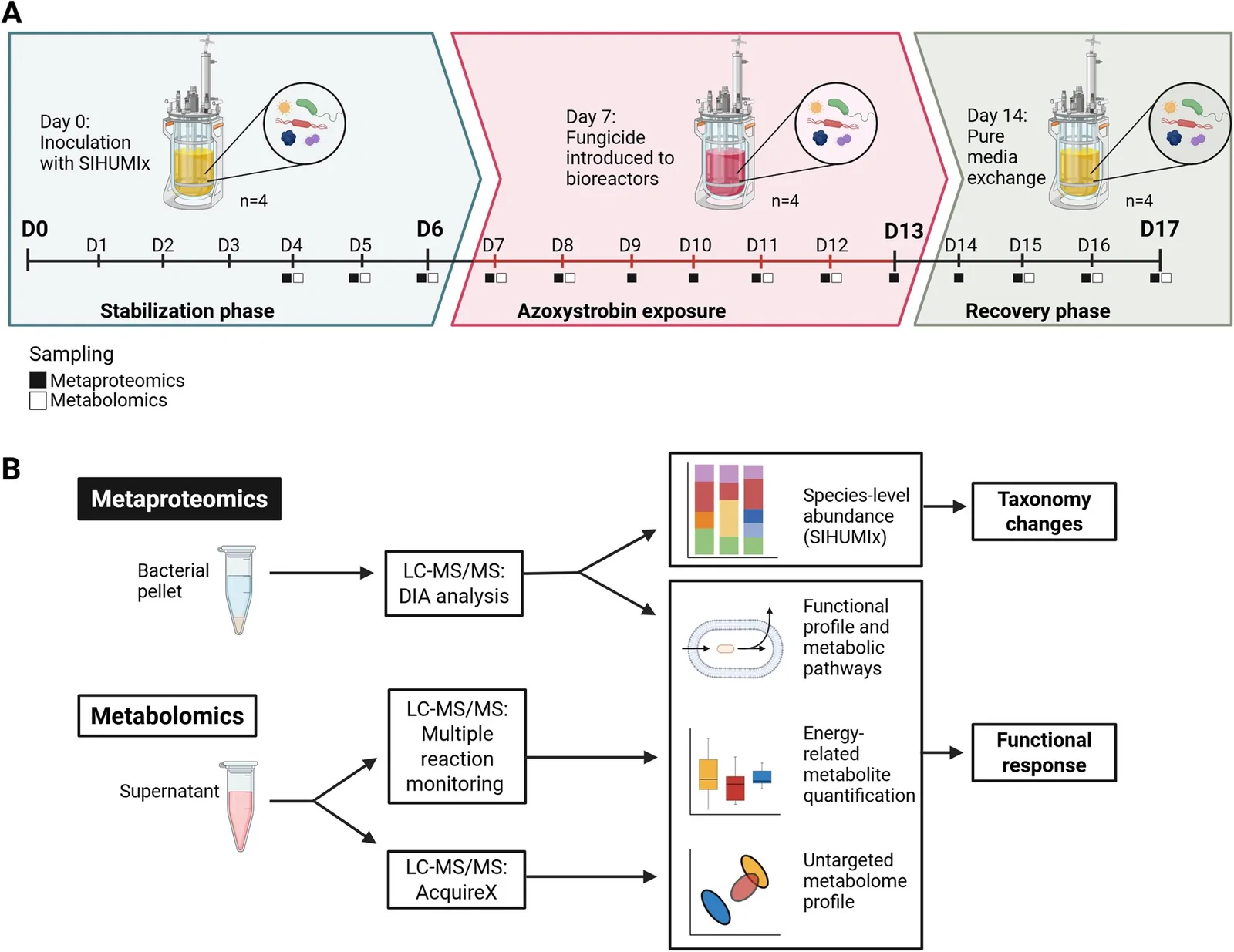
The impact of azoxystrobin on the simplified human microbiota model (SIHUMIx) was investigated using a continuous bioreactor cultivation system. A) Following inoculation and a stabilization period of 6 days, azoxystrobin was introduced to the system at a concentration of 0.0056 mg/ml over the course of 7 days. Chemical washout began at the end of day 13, marking the start of the recovery period. B) Daily samples were taken from day 4 to day 17 for downstream metaproteomics and metabolomics. Adapted from Castañeda-Monsalve et al. (2025). Created in BioRender (Wray 2026a) . https://BioRender.com/ocnmhi4.

The SIHUMIx community has been well-characterized in bioreactor systems, with prior work demonstrating that it reaches compositional and functional stability under defined conditions by day 4 of continuous cultivation (Fig. 1). Under these parameters, community structure based on protein biomass, species abundance profiles, SCFA production, flow cytometry-based fingerprints, intact protein patterns, and terminal restriction fragment length polymorphisms remains consistent through at least 21 days of culture (Schäpe et al. 2019; Krause et al. 2020; Haange et al. 2024). Importantly, previous work has also demonstrated that SIHUMIx is capable of returning to its pre-exposure state following both pulse and press disturbances (Schäpe et al. 2019; Krause et al. 2020). Therefore, the cultivation parameters and stabilization period used in the present study were selected to align with previously validated conditions.

Based on the established stability of SIHUMIx, our experimental design focused on comparing temporal stages within the same bioreactors rather than across separate systems. By using the late stabilization phase (beginning at day 4) as a reference for a steady-state community, this approach minimizes inter-reactor variability and enhances statistical robustness through within-reactor comparisons.

Before inoculation, each of the 8 SIHUMIx strains was revived in 9 ml of Brain Heart Infusion (BHI) medium supplemented with L-cysteine hydrochloride (0.5 g/l), vitamin K–hemin solution (10 ml/l; Becton Dickinson, Heidelberg, Germany), and yeast extract (5 g/l). Cultures were incubated overnight at 37 °C under anaerobic conditions with shaking at 175 rpm. Cell densities were quantified, and an inoculum containing 1 × 10^9^ cells per strain (totaling 8 × 10^9^ cells) was prepared for each bioreactor.

On day 0, SIHUMIx was introduced into 4 sterile 250 ml Multifors 2 bioreactors (Infors, Switzerland), each containing Complex Intestinal Medium (Table S1). Cultures were initially grown at 37 °C in batch mode under continuous stirring at 200 rpm. Anaerobic conditions were established by constant nitrogen gas infusion, and pH was maintained at 6.5 to simulate the distal colon. After 24 h, the system was transitioned to continuous flow mode with a 24 h medium exchange rate, maintaining the same environmental parameters for the remainder of the experiment.

After 6 days of stable cultivation, azoxystrobin was introduced into the SIHUMIx bioreactors at a concentration corresponding to 10% of the Acceptable Daily Intake (ADI), which has been set at 0.2 mg/kg body weight per day (European Food Safety Authority 2010). Assuming an average adult weight of 70 kg and estimating that each bioreactor contains approximately 10% of the bacterial cells typically expelled in daily human feces (∼2 × 10^13^ CFU) (Stephen and Cummings 1980), we calculated the corresponding dose to be 0.0056 mg/ml. To maintain consistent exposure, fresh feed medium was supplemented with azoxystrobin at the same concentration throughout the 7-day exposure period (days 7 to 13).

To monitor recovery following azoxystrobin exposure, the xenobiotic was removed from the feed media at the end of day 13, initiating a 4-day recovery phase. During this period (days 14 to 17), bioreactors were maintained under the same cultivation conditions, but the inflow medium no longer contained azoxystrobin. This exposure timeline was designed to assess the resilience of the microbial community while avoiding biofilm development, which typically begins to occur in this system after day 21. Starting on day 4, daily sampling was performed by collecting four 2 ml aliquots from each bioreactor. Samples were centrifuged at 5,000 × *g* for 10 min to separate supernatants and pellets, which were then stored at −80 °C for downstream analyses.

### Sample preparation for metaproteomic analysis

Bacterial pellet lysis and protein extraction were carried out following the protocol described in (Castañeda-Monsalve et al. 2024). Protein concentrations were quantified using the Pierce 660 nm Protein Assay (Thermo Fisher Scientific, Waltham, Massachusetts, United States). For protein cleanup and proteolytic cleavage using trypsin, a modified version of the Single-Pot Solid-Phase-enhanced Sample Preparation method was applied (Hughes et al. 2019), with detailed steps provided in the Supplementary Methods (Section S2.3). The resulting peptide lysates were transferred to glass vials and stored at −80 °C until subsequent LC–MS/MS analysis.

### LC–MS/MS measurements

Nano LC–MS/MS analyses were performed using a Vanquish Neo nano high-performance liquid chromatography (HPLC) system (Thermo Fisher Scientific) coupled to an Orbitrap Exploris 480 mass spectrometer (Thermo Fisher Scientific) operating in data-independent acquisition (DIA) mode. Detailed settings for the liquid chromatography method and the mass spectrometer are provided in the Supplementary Methods (Section S2.4).

### Spectral library generation for DIA analysis

A spectral library was generated using 490 non-fractionated data-dependent acquisition runs of SIHUMIx samples previously measured in our laboratory. Spectral library construction was carried out using Spectronaut Pulsar (v18.6.231227.55695, Biognosys AG, Schlieren, Switzerland). Protein identification was based on strain-specific protein sequence databases for each of the 8 SIHUMIx species, all retrieved from UniProt Proteomes (www.uniprot.org). The datasets included the following entries: *A. caccae* (*n* = 3,743), *B. thetaiotaomicron* (*n* = 4,782), *B. longum* (*n* = 1,725), *B. producta* (*n* = 5,372), *C. butyricum* (*n* = 4,015), *T. ramosa* (*n* = 3,166), *E. coli* K-12 (*n* = 4,448), and *L. plantarum* (*n* = 3,087). The resulting spectral library contained 1,273,751 fragment ions, 212,981 precursors, and 174,109 peptides, resulting in 12,318 distinct protein groups.

### Metaproteomic DIA data processing

Mass spectrometry data were processed using Spectronaut (Biognosys AG), employing the previously constructed SIHUMIx spectral library with the standard Biognosys default pipeline. Please refer to Supplementary Methods (Section S2.6) for additional information on data processing.

### SCFA analysis for SIHUMIx

SCFA quantification was conducted using a derivatization-based LC–MS/MS method adapted from Han et al. (2015). For details on sample preparation and metabolite quantification, please refer to Supplementary Methods (Section S2.7).

### Untargeted metabolomics

To broadly profile metabolites present in the samples, untargeted metabolomics was applied using an HPLC system paired with an Orbitrap IQ-X mass spectrometer (Thermo Fisher Scientific), utilizing the AcquireX acquisition workflow. Specific details regarding sample handling, experimental conditions, and data acquisition are described in the Supplementary Methods (Section S2.8).

### Statistical analysis of SIHUMIx microbiota and omics data

All statistical analyses related to the SIHUMIx microbiota and associated omics datasets were performed using R (R v4.3.1; R Development Core Team). To account for repeated measurements over time within the same bioreactor, differences across experimental phases were assessed using linear mixed-effects models (LMMs). For each molecular feature (SCFAs, amino acids, proteins), concentration or intensity was modeled as a function of experimental phase (stabilization, exposure, recovery) as a fixed effect, with bioreactor included as a random intercept to account for non-independence of observations within bioreactors. Models were fitted using the lme4 package (Bates et al. 2015). Post-hoc pairwise comparisons between experimental phases were performed on estimated marginal means using Tukey adjustment for multiple testing with the emmeans package (Lenth and Piaskowski 2017). Significance was defined at *P* < 0.05. For analyses of mean relative abundances of individual SIHUMIx species across cultivation phases, statistical comparisons were performed on the mean values per bioreactor for each experimental phase. Because these data summarize phase-level changes rather than temporal measurements, differences were assessed using Kruskal–Wallis (KW) tests, followed by Dunn’s post-hoc tests. Benjamini–Hochberg correction was applied to account for multiple pairwise phase comparisons (*P* < 0.05). Principal component analysis (PCA) was applied to taxonomic, metabolomic, and functional data to visualize group-level variation. Because the data consist of numeric species-level protein abundances rather than compositional sequencing counts, PCA was applied to enable inspection of feature loadings. Statistical significance between groups was tested using permutational multivariate analysis of variance (PERMANOVA) based on Bray–Curtis dissimilarities, implemented with the adonis2 function in the vegan package (Dixon 2003). When the global PERMANOVA was significant, pairwise PERMANOVA comparisons between groups were performed, and *P*-values were adjusted for multiple testing using the Hochberg method.

### Bacterial culturing and preparation for larval zebrafish exposure

Bacterial strains were maintained as glycerol stocks at –80 °C. For experimental use, SIHUMIx strains were reactivated by inoculating BHI broth in sealed Hungate tubes. Cultures were incubated under appropriate conditions, and optical density at 600 nm (OD_600_) was monitored during growth. Once cultures reached the target range for exposure preparations (0.5 to 1.5), they were processed for dilution. To standardize bacterial concentrations across strains, OD_600_ values were first multiplied by 10 and then converted to estimated cells/ml using an OD-to-cell conversion calculator (https://www.chem.agilent.com/store/biocalculators/calcODBacterial.jsp). From these values, the volume of bacterial suspension needed to achieve an exposure concentration of 100 cells/ml in each zebrafish flask was calculated. To generate water-compatible microbial solutions, 1 ml of bacterial culture was transferred to a microcentrifuge tube and centrifuged at maximum speed (∼12,000 × *g*) for 1 min. The supernatant was discarded, and the resulting pellet was resuspended in 1 ml of sterile filter-sterilized (FS)-10% Hanks’ balanced salt solution (HBSS) (heat-inactivated fetal serum in HBSS). This suspension was then added to 249 ml of sterile FS-10% HBSS to produce a 250 ml intermediate stock. Microcentrifuge tubes were rinsed with 1 ml of the final solution and returned to the stock to maximize cell transfer. Exposure water was prepared by diluting the intermediate stock further to reach 100 cells/ml in FS-10% HBSS.

### Zebrafish husbandry

All zebrafish (*Danio rerio*) work conducted at the Helmholtz Center for Environmental Research (UFZ) was approved by the local government authority (Landesdirektion Sachsen, Geschäftszeichen 24-5131/252/7) and adhered to German and European animal protection guidelines. Adults from the in-house wild-type “UFZ-OBI/WIK” zebrafish strain were housed in 27 l glass tanks at a density of approximately 5 fish/l. Adult fish were fed both Zebrafeed dry food (Sparos) and shell-free artemia (Sanders) daily on weekdays, with a daily feeding of artemia on the weekends. Every 1 to 2 weeks, adult zebrafish were bred for embryo collection using breeding trays placed in on-rack glass tanks. Embryos were collected the morning after set up, and fertilized embryos with normal morphology were selected for experimental use using a dissection microscope (Olympus SZx7-ILLT). Water in the recirculating aquaculture system was exchanged approximately 5×/h/tank, resulting in a 10% daily replacement of system water with fresh water. Prior to being recirculated back into the tanks, system water was filtered and sterilized with UV. The lighting conditions followed a 14:10 light:dark cycle, with 8 h of direct overhead light and 6 h of ambient light, stimulating daily light fluctuations. Fish tank water quality was routinely monitored for the following parameters: Water temperature: 26 to 29 °C; pH: 7 to 8; water hardness: 4 to 16°dH; nitrite: 0 to 0.05 mg/l; nitrate: 0 to 50 mg/l; ammonium: 0 to 0.35 mg/l; oxygen saturation: 87% to 91%.

### Generation of microbiome-depleted and SIHUMIx-inoculated larvae

In order to derive the cohort with a depleted microbiome and remain in line with previously published work (Pham et al. 2008; Milligan-McClellan et al. 2011; Melancon et al. 2017), 0 days post fertilization (dpf) embryos were collected in a petri dish containing FS-10% HBSS with a mixture of antibiotics (100 µg/ml ampicillin, 5 µg/ml kanamycin, and 0.25 µg/ml amphotericin B) and incubated at 28 °C for 3 h. Embryos were then pipetted into a 15 ml conical tube, which was transferred into a sterile cell culture hood. The embryos underwent a series of 3 rinses in the antibiotic HBSS, followed by 2 min of submersion in FS-10% HBSS containing 0.5% poly(vinylpyrrolidone)-iodine (PVP-I), a broad-spectrum antimicrobial agent. After being treated with PVP-I, embryos were rapidly rinsed in FS-10% HBSS and then submerged in FS-10% HBSS containing 0.05% bleach for 20 min. A final series of 3 rinses with FS-10% HBSS was performed before the embryos were pipetted into sterile T25 tissue culture flasks (25 ml of FS-10% HBSS, *n* = 30 embryos per flask) in a sterile hood.

**Fig. 2.**
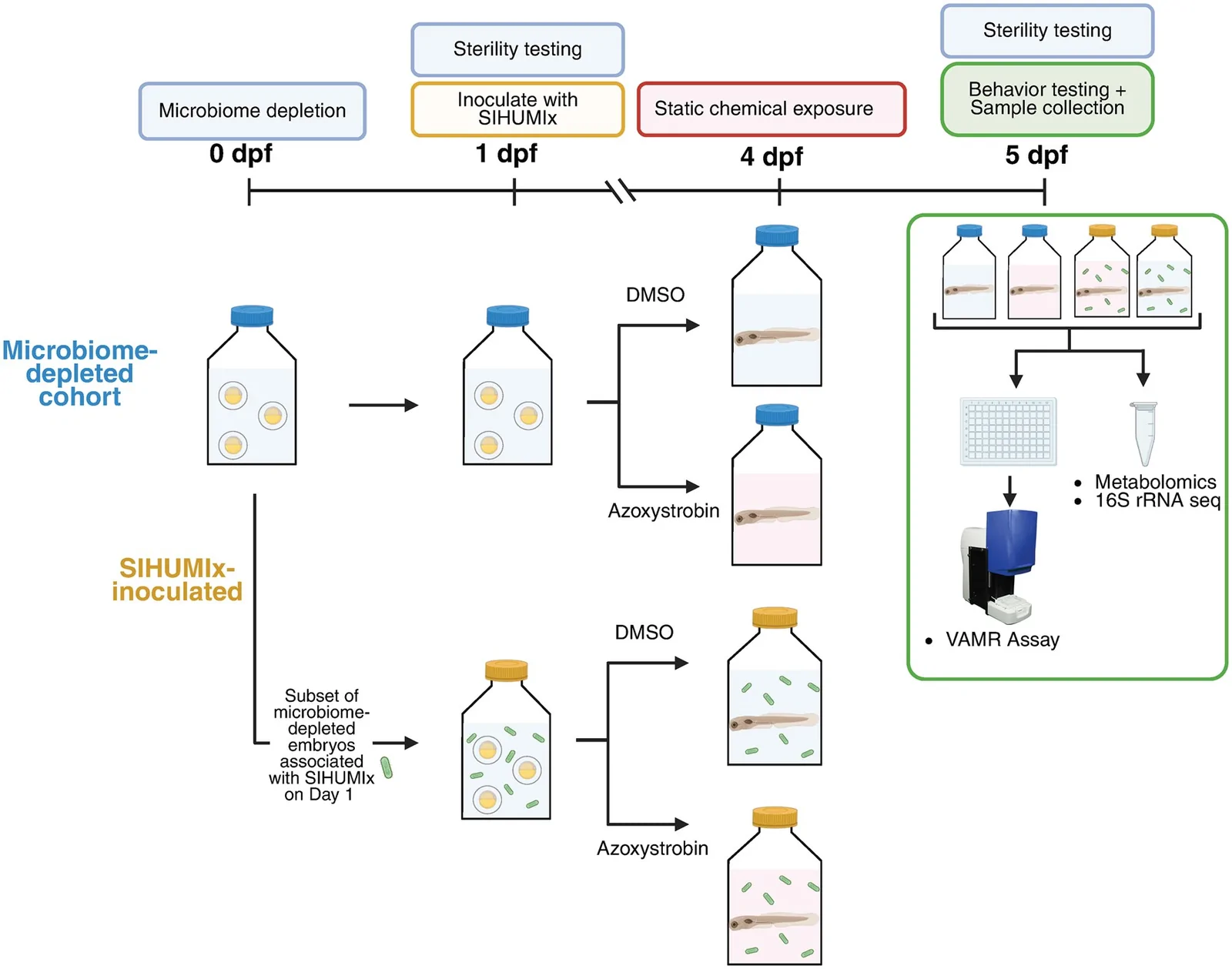
Zebrafish derivation experimental design. Cohort generation and exposure design. At 0 dpf, zebrafish embryos were collected and underwent microbiome depletion (*n* = 30 embryos/flask). Microbiome-depleted larvae were maintained under sterile conditions, whereas a subset of embryos was inoculated with SIHUMIx on day 1 to generate the SIHUMIx-inoculated cohort. At 4 dpf, larvae in both cohorts were statically exposed to azoxystrobin (0.6 or 1.68 μM) or the vehicle control (0.4% DMSO). At 5 dpf, larvae were plated for behavior assessment with the Visual Acoustic Motor Response (VAMR) assay, with remaining larvae collected for 16S rRNA sequencing or metabolomics analyses. Created in BioRender (Wray 2026b). https://BioRender.com/ymgg921.

At 1 dpf, roughly 50% of the microbiome-depleted embryos were inoculated with the SIHUMIx to generate the SIHUMIx-inoculated cohort (Fig. 2). In line with previous work, larvae were exposed to media with microbes at a density of 100 cells/ml (Phelps et al. 2017; Weitekamp et al. 2021). In this study, we inoculated with SIHUMIx (solution prepared as outlined in the “Bacterial culturing and preparation for larval zebrafish exposure” Section), maintaining the same colonization density. Flasks from both cohorts (microbiome-depleted and SIHUMIx-inoculated) were then incubated at 28 °C with a 14:10 h light:dark cycle. All flasks were maintained in a static environment without media change through 3 dpf. At 4 dpf, flasks were exposed to varying concentrations (0.6 or 1.68 μM) of azoxystrobin or the vehicle control (0.4% dimethyl sulfoxide [DMSO]). Dead embryos were removed during the media change at 1 dpf and the chemical exposure at 4 dpf, and a 1 ml sample of media was taken from each sterile flask at 1 and 5 dpf on the day of behavior testing. Media samples were then used for basic microbiological approaches to confirm the microbiome-depleted status of the larvae.

### Sterility testing of microbiome-depleted flasks

At 1 dpf, media sterility was assessed by inoculating tryptic soy agar (TSA) plates (Sigma #22091) with 10 µl of media from each flask. The plates were then incubated at 28 °C under aerobic conditions and monitored for contamination for a minimum of 7 days. Flasks were excluded from the study if any visible growth appeared on the TSA plates. Flasks with microbiome-depleted larvae were routinely monitored for biofilms, and any exhibiting microbial growth were also excluded from the study. On the day of behavior testing, an additional sterility check was performed by inoculating 100 µl of media from each microbiome-depleted cohort flask into tubes containing Nutrient Broth (Sigma #70122), BHI broth (Sigma #53286), or Sabouraud Dextrose Broth (Sigma #S3306). The broth tubes were then incubated under aerobic conditions at 28 °C for at least 7 days following the conclusion of the experiment. Larvae were deemed microbiome-depleted only if TSA plates and all 3 broth types showed no bacterial growth and if no biofilm was detected after the designated incubation period.

### Chemical preparation for larval exposures

Azoxystrobin (Chemical Abstracts Service Registry No.: 131860-33-8, Catalog No. 43854) was purchased from Sigma-Aldrich. Stock solutions (30 mM) were prepared by dissolving the neat chemical into anhydrous DMSO (Sigma-Aldrich), and aliquots were stored at −80 °C in 2 ml amber glass vials. Single-use stock solution aliquots were thawed for each experiment and discarded following exposure. For range-finding experiments aimed at determining the highest concentration with no observable effects on morphology, 250× working solutions were prepared from a thawed stock. In line with previous work, a 250× stock plate was generated by performing semi-logarithmic serial dilutions of azoxystrobin in DMSO within a 96-well polycarbonate microtiter plate (Gaballah et al. 2020; Gutsfeld et al. 2024). For experiments requiring sterility, 100 μl of the 30 mM azoxystrobin stock was added to 10 ml of Hanks’ Balanced Salt Solution (HBSS) to create an intermediate working solution that could then be filtered through a 0.2 μm polyethersulfone membrane into a sterile 15 ml conical tube before use.

### Study design for zebrafish larvae and chemical exposures

#### Range-finding exposures

Range-finding exposures were conducted to determine the no observed effects concentration (NOEC) for azoxystrobin following a 24 h exposure starting at 4 dpf. Embryos were exposed by adding 1.6 μl of 250-fold working solution from the stock plate, resulting in a concentration range from 112.8 to 0.6 µM. The exposure design was selected such that the concentration 13.8 μM, which was used in the SIHUMIX experiments (10% ADI), directly corresponded to one of the tested concentrations. All groups, including the vehicle control, contained a final concentration of 0.4% DMSO. At 5 dpf, larvae were assessed for morphological abnormalities and mortality, and the NOEC for this exposure paradigm (1.68 µM) was selected as the highest concentration for use in future experiments (Fig. S1).

#### Exposing flasks

At 4 dpf, larvae in both cohorts were exposed to either azoxystrobin or 0.4% DMSO. Exposures for the microbiome-depleted larvae were conducted under sterile conditions, in which the microbe-free intermediate working solution (prepared as described under “Chemical preparation for larval exposures” Section) was added to a subset of the flasks to achieve final concentrations of 1.68 and 0.6 µM azoxystrobin. Vehicle control flasks received 100 μl of DMSO to achieve a final concentration of 0.4% DMSO, and additional DMSO was added to azoxystrobin-exposed flasks to maintain this concentration across all experimental groups. Once these sterile exposures were completed, the same solutions were used to expose the SIHUMIx-inoculated cohort under standard laboratory conditions. In total, there were 10 replicates per concentration per microbial colonization cohort (60 flasks total: 30 for each colonization status, with 10 flasks per exposure group: 1.68 µM azoxystrobin, 0.6 µM azoxystrobin, or 0.4% DMSO).

### Automated behavior assay

At 5 dpf, single larvae from each cohort and concentration were plated from the flasks into sterile 96-well clear squared polystyrene plates with 400 µl of medium per well. Microbiome-depleted larvae were plated on the sterile bench, sealed with Microseal A film (Biorad MSA5001), and then removed from the sterile bench, where the SIHUMIx-inoculated larvae were plated into the remaining wells. After plating, the plates were put into a paper box and set to incubate in the dark for a minimum of 2 h at 28 °C before behavior assessments were performed.

The Visual and Acoustic Motor Response (VAMR) behavior assay utilized was based on previous work (Leuthold et al., 2025; Herold et al. 2025). Following the period in the box, the plates were transferred to the Zebrabox behavior apparatus (ViewPoint), continuously shielded from infrared light. To measure locomotor activity (changes in pixel intensity/second), videos were captured at 25 frames per second with ZebraLab video tracking software (ViewPoint) in “quantization mode” (sensitivity threshold = 15). This assay utilized 4 different stimulus modalities, encompassing visual light exposure ranging from 0 lux (dark condition) to 13.5 klux (light condition; measured with an LI-250 light meter from LI-COR), as well as low-volume (65 dB) and high-volume (75 dB) acoustic stimuli delivered at a frequency of 300 Hz. The assay started with a 21 min acclimation period in darkness (0 lux), followed by 10 min of light exposure before transitioning back to darkness for the remainder of the assay. After the light had been off for 20 min, the acoustic stimulus phase started. This portion of the assay was split into multiple segments: First, 5 low-volume acoustic stimuli played at 1-min intervals. After a 1-min inter-endpoint interval, the next segment introduced 5 high-volume acoustic stimuli, again spaced by 1 min between each stimulus. Another 1-min inter-endpoint interval preceded the habituation phase, which consisted of 5 bouts delivered 1 min apart, with each bout containing 30 high-intensity acoustic stimuli played at 1-s intervals. Following a 3-min inter-endpoint interval, a final set of 5 high-volume acoustic stimuli was delivered, each 1 min apart.

### Processing of automated behavior data

Motor activity data (changes in pixel intensity/s/well) were analyzed using custom workflows (Herold 2024; Leuthold 2025) in KNIME (v4.6.3, KNIME AG, www.knime.com) that incorporated R scripts (R Core Team 2023) for statistical analysis and visualization. The analysis utilized the ggplot2 (Wickham 2016), RColorBrewer (Neuwirth 2022), and ComplexHeatmap (Gu 2022) packages. To quantify motor activity, cumulative pixel intensity changes per second were calculated per well for each endpoint across the full assay. Baseline motor activity was recorded during the initial 21-min dark acclimation period and quantified as the average motor activity per second, divided into four 5-min intervals (BSL1, BSL2, BSL3, and BSL4). Following the initial dark-to-light transition, motor activity was divided into 2 phases: A rapid, 1-s visual startle response (VSR1), followed by a 9-min 59-s period of basal motor activity (VMR1). After the subsequent light-to-dark transition, motor activity was divided into 5 distinct phases: First, a 1-s visual startle response (VSR2), then a progressive increase in motor activity over the following 4-min 59-s period (VMR2), and lastly, a reduction in motor activity that returned to baseline levels, segmented into 3 5-min intervals (VMR3, VMR4, and VMR5). Motor activity was then assessed in response to 5 low-intensity (ASR1) and 5 high-intensity (ASR2 and ASR3) 1-s acoustic stimuli, each separated by 1-min intervals. The motor responses to these stimuli were averaged across 5 trials at each intensity level. The inter-stimulus intervals (ISIs) were calculated by measuring the motor activity per second between the aforementioned acoustic startle responses (ASR1-3), with ISI1, ISI2, and ISI3 representing the motor activity between each of these responses. To assess the potentiation of habituation, 5 consecutive bouts of habituation were delivered 1 min apart, each consisting of 30 high-intensity acoustic stimuli (1-s/stimulus, with a 1-s interval between tones). The first bout is taken as endpoint ASH1, where motor responses to stimuli were summarized into a single metric reflecting acoustic startle habituation. To quantify habituation, motor activity during the final 10 stimuli was compared with the average activity during both the initial and final sets of 10 stimuli combined. This adjustment worked to account for differences in individual baseline startle activity (Lloyd et al. 2014). The potentiation of habituation (ASH1/5) was determined by calculating the ratio of motor activity during the final bout (ASH5) to the sum of the motor activity during both the initial (ASH1) and final (ASH5) bouts. Total motor activity across all 5 habituation bouts was calculated as the acoustic startle habituation sum (ASHsum), whereas the inter-bout interval (IBI) reflected the motor activity per second between each of the 5 habituation bouts. Following habituation trials, a 3-min rest period (IEI3) was given. After this, 5 high-intensity acoustic stimuli (1-s duration, with 1-min spacing between each; ASR3) were presented. Motor responses were averaged across trials and scaled to the average responses from ASR2 and ASR3 to assess memory retention (ASR2/3) post habituation training.

### Statistical analyses for behavior data

Statistical analyses were performed on all 25 endpoints, encompassing baseline activities (BSL1 to BSL4), visual startle responses (VSR1 and VSR2), visual motor responses (VMR1 to VMR5), acoustic startle responses (ASR1 to ASR3), acoustic ISIs (ISI1 to ISI3), inter-acoustic endpoint intervals (IEI1 to IEI3), acoustic startle habituation (ASH1), potentiation of habituation (ASH1/5), acoustic startle habituation sum (ASHsum), IBI, and memory retention (ASR2/3). Given the diverse behavioral metrics and their primarily nonparametric probability distributions, a nonparametric bootstrapping method was applied. This approach, free from model assumptions (Kulesa et al. 2015), used the absolute difference in medians between untreated controls and each treatment as the test statistic for a 2-sided test. First, an N × B matrix was generated, where N represented the number of observation samples, and B (set to 10,000) represented the number of bootstrap samples. Each column contained a bootstrap resample (with replacement) of the observed data. Next, the bootstrap test statistic (absolute difference in medians) was computed for each resample while accounting for the number of observations per group. Finally, the *P*-value was determined as the proportion of bootstrap test statistics that met or exceeded the observed test statistic. To control for family-wise type I error rates, a Benjamini–Hochberg correction was applied to adjust for multiple comparisons. For adjusted *P*-values (*P*_adj_) <0.05, the results were considered significant. Both raw and adjusted *P*-values for all behavioral endpoints are provided in the associated Zenodo data repository (Wray et al. 2026). To estimate 95% confidence intervals for the median of each 1-s time bin, 1,000 bootstrap resamples (with replacement) were drawn from the observed data, and the percentile method was used to define the lower (2.5th percentile) and upper (97.5th percentile) confidence interval bounds.

### Sample preparation for downstream analyses

As outlined in the study design for zebrafish larvae and chemical exposures (“Study design for zebrafish larvae and chemical exposures” Section), zebrafish larvae from the microbiome-depleted and SIHUMIx-inoculated cohort were treated with either 1.68 µM azoxystrobin, 0.6 µM azoxystrobin, or 0.4% DMSO (vehicle control). At 5 dpf, flasks were placed on ice for a minimum of 1 h to anesthetize the zebrafish larvae. From each flask, 2 separate pools of 10 whole larvae were collected, transferred to 1.5 ml tubes (Eppendorf Tubes), and kept on ice. After removing the remaining liquid, samples were flash-frozen in liquid nitrogen and stored at −80 °C until further processing.

### 16S rRNA of whole zebrafish larvae and flask media

#### Sample preparation

For 16S rRNA sequencing of larval zebrafish, 7 to 8 flasks per treatment group were sampled, where each flask was considered to be the experimental unit. From each selected flask, 10 larvae were pooled into a single 1.5 ml tube (Eppendorf Tubes) for DNA extraction. No pooling was performed across flasks, and each flask served as an independent biological replicate. Bacterial DNA was extracted from whole larvae using the FastDNA SPIN Kit for Feces (MP Biomedicals, United States) with modifications outlined in the Supplemental Methods (Section S2.20). Although whole-larvae extraction inherently captures both gut and non-gut microbial communities, this approach was selected to enable higher-throughput microbial and behavioral analyses across all treatment groups. For sequencing of the SIHUMIx inoculum (*n* = 3) and media from SIHUMIx-inoculated flasks exposed to 1.68 µM azoxystrobin, 0.6 µM azoxystrobin, or 0.4% DMSO (*n* = 3 per condition), bacterial DNA was extracted using Chelex 100 resin (Bio-Rad Laboratories, United States) following the manufacturer’s standard protocol for bacterial DNA extraction.

#### Gene sequencing and statistical analyses

The extracted DNA samples from zebrafish larvae, media, and SIHUMIx inoculum described in the “Sample preparation” Section were sent to GENEWIZ Germany GmbH (Leipzig, Germany) for amplification and sequencing of the bacterial 16S rRNA gene. PCR amplicons of the V3 to V4 regions of the bacterial 16S rRNA gene were prepared using the forward and reverse primers 341F and 785R (Thijs et al. 2017). Sequencing libraries were prepared from 100 ng of DNA according to the Illumina protocol. Dual index adapters for the sequencing were attached using the NEBNext Multiplex Oligos for Illumina. The final concentration of the libraries was 2 nM after pooling. We sequenced triplicates of samples from all the studied parameters (16S rRNA, *n* = 58).

The sequencing data were analyzed using QIIME2 v2023.5 (Bolyen et al. 2019). First, the raw sequence reads were demultiplexed and quality-filtered (*q*-score ≥25) using the q2‐demux plugin, followed by denoising with DADA2 (Callahan et al. 2016) (via q2‐dada2). The 16S forward and reverse sequences were trimmed at 250 and 245 bp, respectively. All amplicon sequence variants (ASVs) were aligned with mafft (Katoh et al. 2002) (via q2‐alignment), and then maximum-likelihood trees were constructed using FastTree2 (Price et al. 2010) (via q2‐phylogeny). We chose ASV-based methods over operational taxonomic units (OTUs) approaches to allow for better comparability across studies and to limit the effect of spurious taxa on diversity indices (Reitmeier et al. 2021; Chiarello et al. 2022). Taxonomic assignment of bacterial ASVs was carried out using the q2‐feature‐classifier (Bokulich et al. 2018) and the classify-sklearn Naïve Bayes taxonomy classifier against the Greengenes2 “99% OTU reference sequences” (McDonald et al. 2024). In the 16S rRNA sequencing analysis, the ASV abundance tables were filtered with total-frequency-based filtering based on 95% sequence identity (via q2-feature-table summarize) and rarefied at 1,560 sequences to ensure equal sampling depth and sorting in the maximum number of samples for diversity analyses. For taxonomic composition summaries and visualization, ASVs were further filtered to retain those detected in at least 10% of samples within each experimental group and that contributed at least 1% relative abundance to any of the sample totals (Phelps et al. 2017; Weitekamp et al. 2021). For figures specifically depicting the SIHUMIx consortium members, the dataset was restricted to ASVs taxonomically corresponding to the defined consortium members.

### SCFA, amino acid, and biogenic amine quantification in zebrafish

#### Metabolomics

Samples were generated as outlined in the “Sample preparation for downstream analyses” Section with 6 replicate pools per concentration and colonization status. Each sample was mixed with 50 µl of extraction solvent acetonitrile (ACN):H_2_O (1:1, v/v/v) and homogenized using a TissueLyser II (30 Hz, 10 min; Retsch Qiagen). After centrifugation (2 min, 14,000 rpm), 10 and 20 µl were used for amino acid and SCFA derivatization, respectively. SCFA quantification followed the same derivatization-based LC–MS/MS method used for SIHUMIx (Supplemental Methods, Section S2.7; Han et al. 2015). For derivatization of amino acids and biogenic amines, the supernatant was evaporated to dryness (SpeedVac, Eppendorf). The samples were resuspended in 50 µl 5% phenyl isothiocyanate (PITC) in ethanol:H_2_O:pyridine (1:1:1, v/v/v) and incubated for 25 min at RT. Subsequently, samples were dried to remove excess PITC and resuspended in 10 µl 5 mm ammonium acetate in methanol. After incubation (10 min, 14,000 rpm), 90 µl H_2_O:ACN + 0.2% formic acid were added.

Prior to measurement, 10 µl of each derivative was injected onto a Waters Acquity UPLC system coupled online with a QTRAP 5000 mass spectrometer (Sciex, Framingham, United States). Chromatographic separation was achieved with an Agilent Zorbax Eclipse XDB-C18 column (3.5 µm, 3.0 × 100 mm) using a constant flowrate of 0.5 ml/min and water + 0.2% formic acid and ACN + 0.2% formic acid as mobile phases A and B, respectively. The linear LC gradient was as follows: 0 to 0.5 min at 0% B, 0.5 to 4 min 0% to 70% B, 4 to 5.3 min 70% B, 5.3 to 5.4 min 70% to 0% B, 5.4 to 7.3 min 0% B, and the QTRAP was set up to positive ionization mode.

For identification and quantitation, a scheduled multiple reaction monitoring method was used, with specific transitions for every SCFA, amino acid, and biogenic amine. External calibration curves for each metabolite were measured for regression. Peak areas of all samples and standards were determined in SciexOS Software (v. 3.0.0., Sciex).

#### Statistics for zebrafish metabolomics and pathway identification

To determine the appropriate statistical tests for assessing amino acids, biogenic amines, and SCFA levels across treatment groups in larval zebrafish, data distributions were first evaluated for normality using the Shapiro–Wilk test in R (v4.3.1). All compounds were subsequently analyzed in MetaboAnalyst6.0 (Pang et al. 2024) according to their distribution. For normally distributed compounds, the effects of azoxystrobin exposure, microbial colonization status, and their interactions were tested using the Statistical Analysis [metadata table] module with the 2-way ANOVA (ANOVA2) option. When significant effects were observed (*P* < 0.05), post-hoc comparisons were performed in R using Tukey’s honestly significant difference (HSD) test. For non-normally distributed compounds, each factor (azoxystrobin exposure or microbial colonization status) was analyzed separately using the Statistical Analysis [1 factor] module with the non-parametric ANOVA (KW, *P* < 0.05). When significant effects were observed for a metabolite, post-hoc pairwise comparisons were conducted in R using Dunn’s test with Benjamini–Hochberg correction for multiple testing.

To identify metabolic pathways potentially impacted by treatment, statistically significant compounds (*P* < 0.05) identified from both the 1- and 2-way ANOVAs were carried forward for pathway enrichment using the MetaboAnalyst Pathway Analysis module with the *Danio rerio* (zebrafish) KEGG library (Supplemental Methods, Section S2.21b). Pathways with an impact score of zero were excluded from interpretation.

## Results

### Azoxystrobin exposure changes proteome-weighted SIHUMIx community structure

**Fig. 3.**
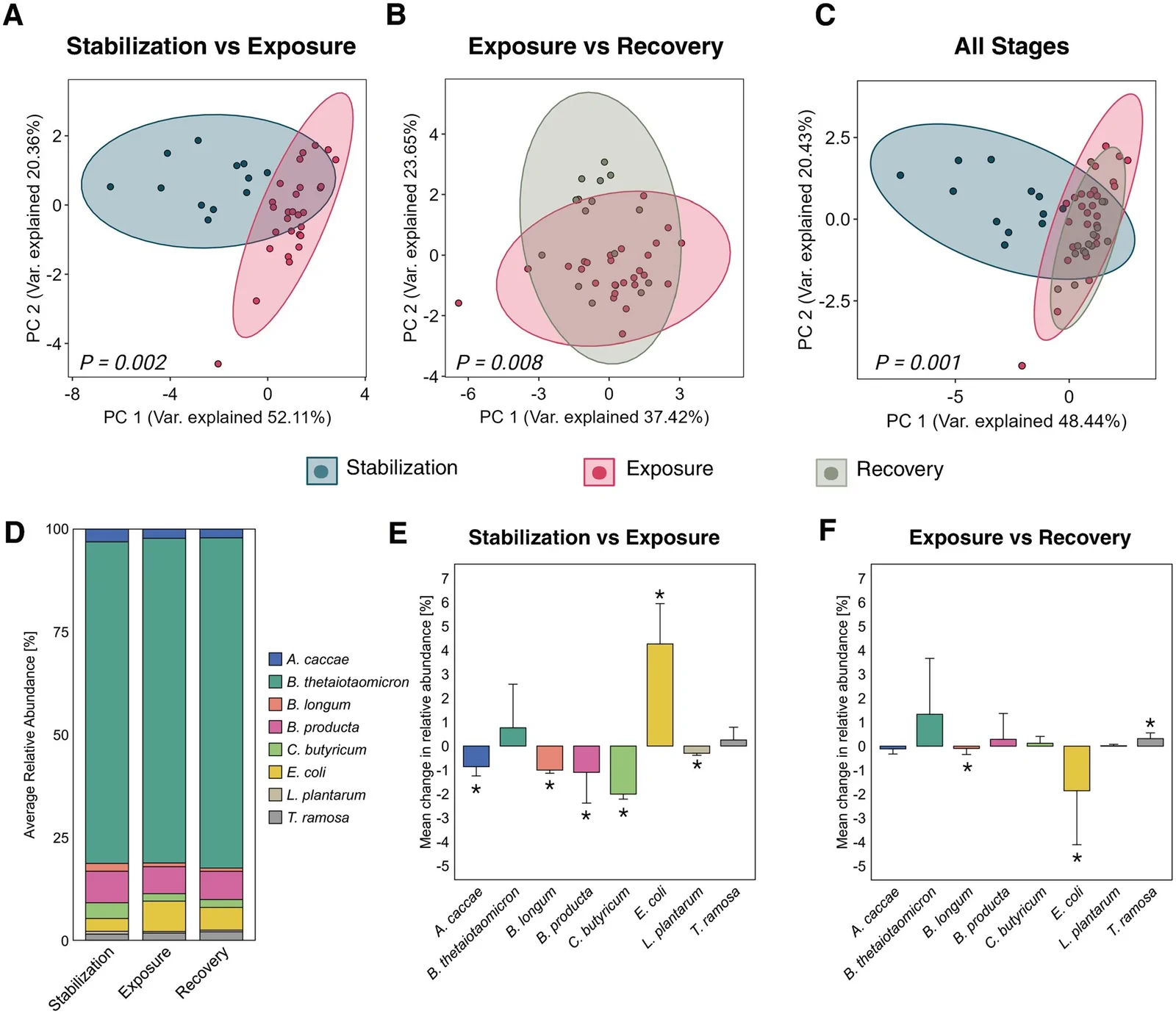
Effect of azoxystrobin on SIHUMIx community composition based on protein biomass. Structural changes in SIHUMIx were monitored throughout the experimental phases. PCA of species’ relative protein biomass contributions comparing A) stabilization to azoxystrobin exposure, B) exposure to recovery, and C) across all phases combined; statistical significance assessed via PERMANOVA. D) Mean relative abundances of species across cultivation phases. E and F) Variations in relative protein abundance of individual SIHUMIx species between stabilization and exposure, and between exposure and recovery. Error bars denote standard deviation; statistically significant differences were determined by KW test followed by Dunn’s test with Benjamini–Hochberg correction (*P* < 0.05) and are indicated with asterisks.

Microbial composition of the SIHUMIx consortium was analyzed based on the relative abundance of species-specific proteins identified by metaproteomics, yielding a proteome-weighted measure of community structure (Kleiner et al. 2017). During the stabilization phase, this community structure based on protein biomass was consistent with previously reported steady-state SIHUMIx species abundance profiles (Schäpe et al. 2019; Krause et al. 2020; Haange et al. 2024). After evaluating protein abundance-based profiles of SIHUMIx species exposed to azoxystrobin for 7 days, followed by a recovery period, clear shifts in proteome-weighted community structure were observed. PCA indicated significant differences between the stabilization and exposure phases (PERMANOVA, *P* = 0.002) (Fig. 3A), as well as between exposure and recovery (PERMANOVA, *P* = 0.008) (Fig. 3B). Moreover, the stabilization and recovery phases also differed significantly (PERMANOVA, *P* = 0.002), suggesting that the microbial community did not return to its pre-exposure configuration.

All SIHUMIx members were detected during the entire experiment (Fig. 3D). Among them, *E. coli* was the only species that significantly increased in relative protein biomass contribution during exposure compared with stabilization (Dunn’s, *P* < 0.001). Conversely, *C. butyricum*, *B. producta*, *A. caccae*, *L. plantarum*, and *B. longum* all decreased proteomic contributions significantly during the exposure phase (Dunn’s, *P* < 0.05 for all) and remained at lower levels than stabilization during recovery (Fig. 3E). Additionally, *T. ramosa* displayed a slight but significant increase in relative protein abundance during the recovery phase (Dunn’s, *P* = 0.003) (Fig. 3F), and the relative abundance of *B. thetaiotaomicron* showed no significant changes during the exposure and recovery stages.

### Azoxystrobin alters metabolic pathways of the SIHUMIx community

**Fig. 4.**
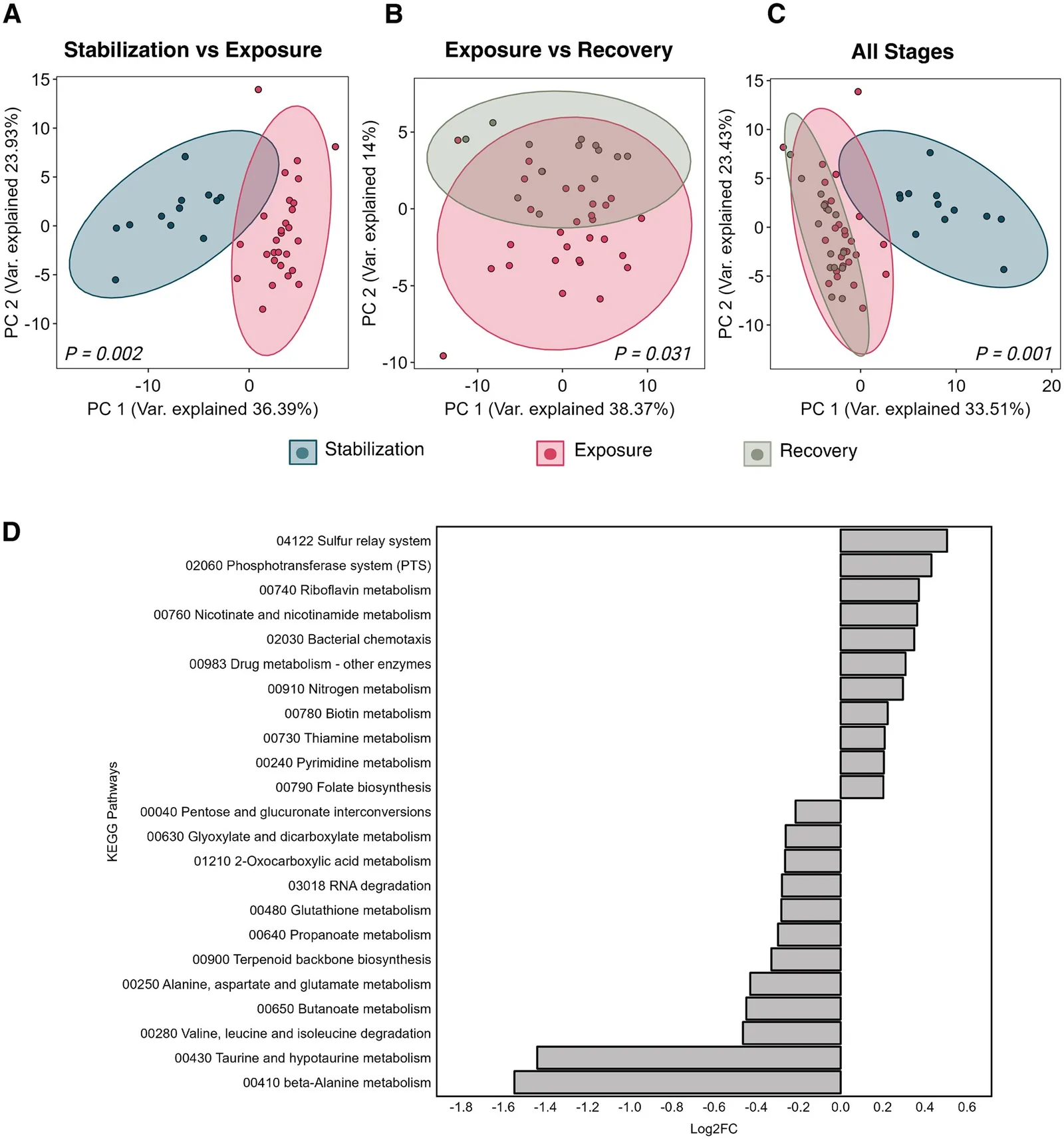
Azoxystrobin alters functional pathways in SIHUMIx. Functional profiling of SIHUMIx following azoxystrobin exposure was performed. PCA of pathway relative abundances comparing A) stabilization to exposure, B) exposure to recovery, and C) across all experimental stages combined; significance assessed using PERMANOVA. D) Key pathways were identified by comparing stabilization and exposure phases, applying a Log2 fold-change cutoff of ±0.2 and statistical significance determined by the KW test (*P* < 0.05).

To characterize the functional shifts in SIHUMIx under azoxystrobin exposure, proteins were grouped according to KEGG metabolic pathways. From a total of 5,544 protein groups detected, 3,652 could be assigned to KEGG orthologs, covering 84 distinct pathways. Only pathways meeting a minimum threshold of 15% functional coverage and at least 5 proteins were retained for analysis. PCA of pathway abundances revealed clear separation between the stabilization, exposure, and recovery phases (PERMANOVA, *P* = 0.001) (Fig. 4A to C), indicating distinct functional profiles across conditions. To highlight the pathways most strongly affected, additional filtering was applied using a KW test (*P* < 0.05) combined with a Log2FC threshold of ±0.2, focusing on comparisons between the stabilization and exposure phases.

A total of 23 significant metabolic pathways were identified (KW test, *P* < 0.05) when comparing the stabilization and exposure phases (Fig. 4D). Of these, 11 pathways were upregulated, with the strongest increases observed in the Sulfur relay system (Log2FC = 0.51), the Phosphotransferase system (PTS) (Log2FC = 0.43), and Riboflavin metabolism (Log2FC = 0.37). Additional enrichment was detected in nicotinate and nicotinamide metabolism (Log2FC = 0.36), Bacterial chemotaxis (Log2FC = 0.35), and Drug metabolism (Log2FC = 0.31). Several essential biosynthetic processes, including Nitrogen metabolism (Log2FC = 0.30), Biotin metabolism (Log2FC = 0.22), Thiamine metabolism (Log2FC = 0.21), Pyrimidine metabolism (Log2FC = 0.21), and Folate biosynthesis (Log2FC = 0.20), were also significantly increased.

Conversely, 12 pathways were downregulated, with the most pronounced decreases observed in beta-alanine metabolism (Log2FC = −1.55) and taurine and hypotaurine metabolism (Log2FC = −1.44). Several central carbohydrate and energy-related processes were consistently reduced, including butanoate metabolism (Log2FC = −0.45), propanoate metabolism (Log2FC = −0.30), and pentose and glucuronate interconversions (Log2FC = −0.21). Key metabolic nodes such as glyoxylate and dicarboxylate metabolism (Log2FC = −0.26), 2-oxocarboxylic acid metabolism (Log2FC = −0.26), and terpenoid backbone biosynthesis (Log2FC = −0.33) were also downregulated. Importantly, multiple amino acid pathways were suppressed, including alanine, aspartate, and glutamate metabolism (Log2FC = −0.43) and valine, leucine, and isoleucine degradation (Log2FC = −0.46). Additionally, reductions in RNA degradation (Log2FC = −0.28) and glutathione metabolism (Log2FC = −0.28) were also observed.

### Changes in SCFAs and associated pathways following azoxystrobin exposure

SCFAs serve as central indicators of bacterial energy metabolism. Variations in their levels, together with changes in the abundance of proteins linked to the corresponding pathways, can provide insights into possible stress responses triggered by azoxystrobin exposure. Across the sampled time points, SCFAs and lactate were quantified in the culture supernatants, and their concentrations were compared between the stabilization, exposure, and recovery phases.

**Fig. 5.**
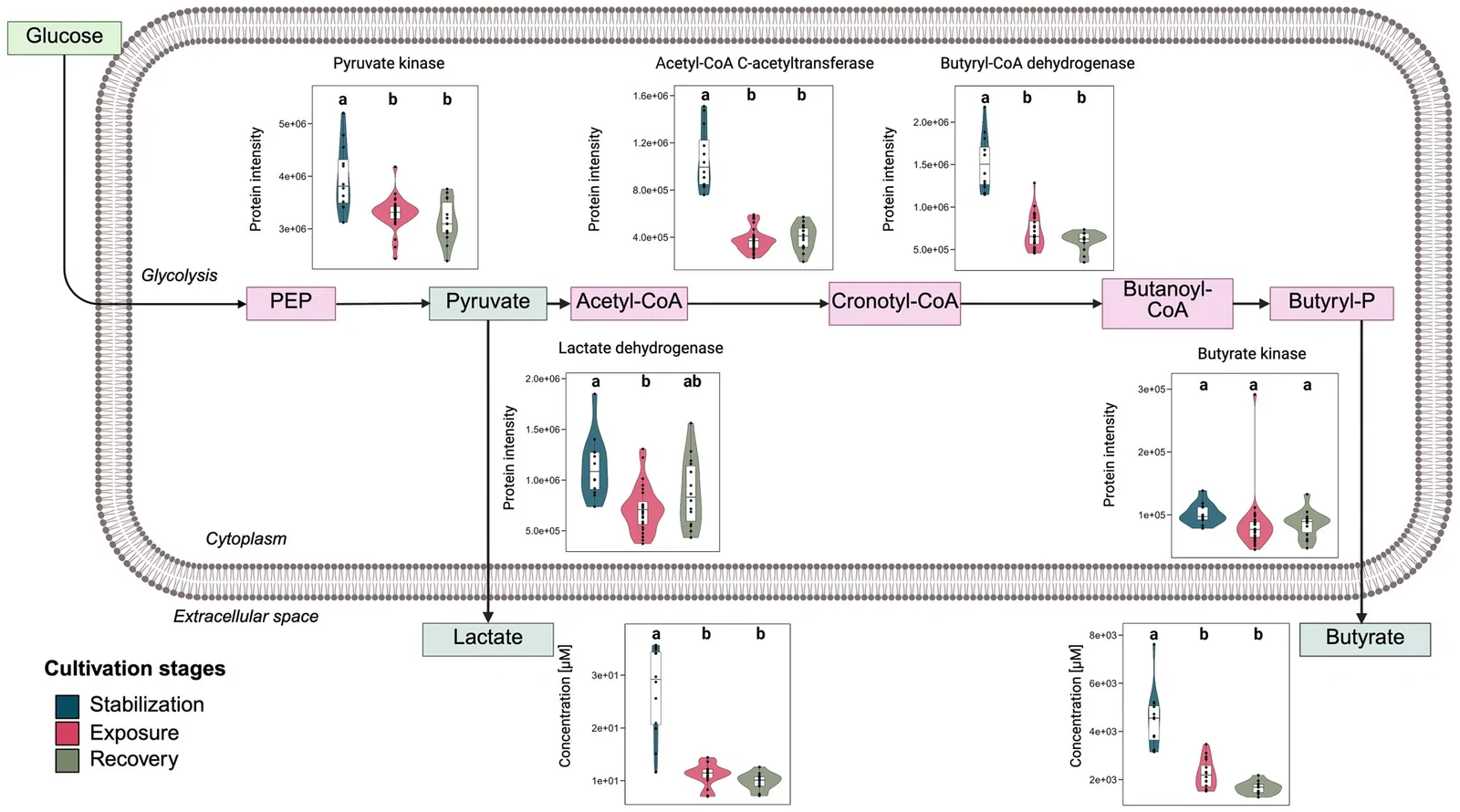
Effects of azoxystrobin on bacterial energy metabolism pathways and related metabolites. Overview of the impact of azoxystrobin on the pyruvate and butyrate metabolism pathways, illustrated with a schematic of a generalized bacterial cytoplasm. Intensities of proteins associated with these pathways are shown across the stabilization, exposure, and recovery phases. In the extracellular compartment, concentrations (µM) of lactate and butyrate are displayed for each phase. Different letters above the plots indicate statistically significant differences based on post-hoc comparisons (LMM followed by Tukey-adjusted pairwise comparisons, *P* < 0.05). Each point represents an individual sample from the 4 bioreactors per cultivation phase. Created in BioRender (Wray 2026c). https://BioRender.com/guzkq04.

Among the main 3 SCFAs: acetate, butyrate, propionate, plus lactate, only butyrate (LMM, *P* < 0.001) and lactate (LMM, *P* < 0.001) showed significant differences between stabilization and exposure. During the recovery period, butyrate and lactate concentrations remained low, similar to those observed under exposure (Fig. 5). To further examine the molecular mechanisms underlying these shifts, we assessed the protein groups mapped to KEGG pathways 00620 (pyruvate metabolism) and 00650 (butyrate metabolism) (Fig. S4). The combined intensity of proteins associated with butyrate metabolism decreased significantly during exposure compared with stabilization (LMM, *P* < 0.001). In recovery, the signal increased again, differing significantly from exposure (LMM, *P* = 0.02), though not reaching the levels detected in stabilization. In contrast, combined proteins assigned to pyruvate metabolism showed no significant change during exposure, but their abundance declined significantly during recovery (LMM, *P* = 0.003). A more detailed inspection of individual enzymes revealed that pyruvate kinase (LMM, *P* < 0.001) was strongly reduced during exposure and remained low after the compound was withdrawn. A similar pattern was observed for lactate dehydrogenase (LMM, *P* < 0.001). Within the butyrate pathway, acetyl-CoA C-acetyltransferase (LMM, *P* < 0.001) and butyryl-CoA dehydrogenase (LMM, *P* < 0.001) were also significantly suppressed following azoxystrobin treatment (Fig. 5).

### Changes in metabolite profile following azoxystrobin exposure in SIHUMIx

**Fig. 6.**
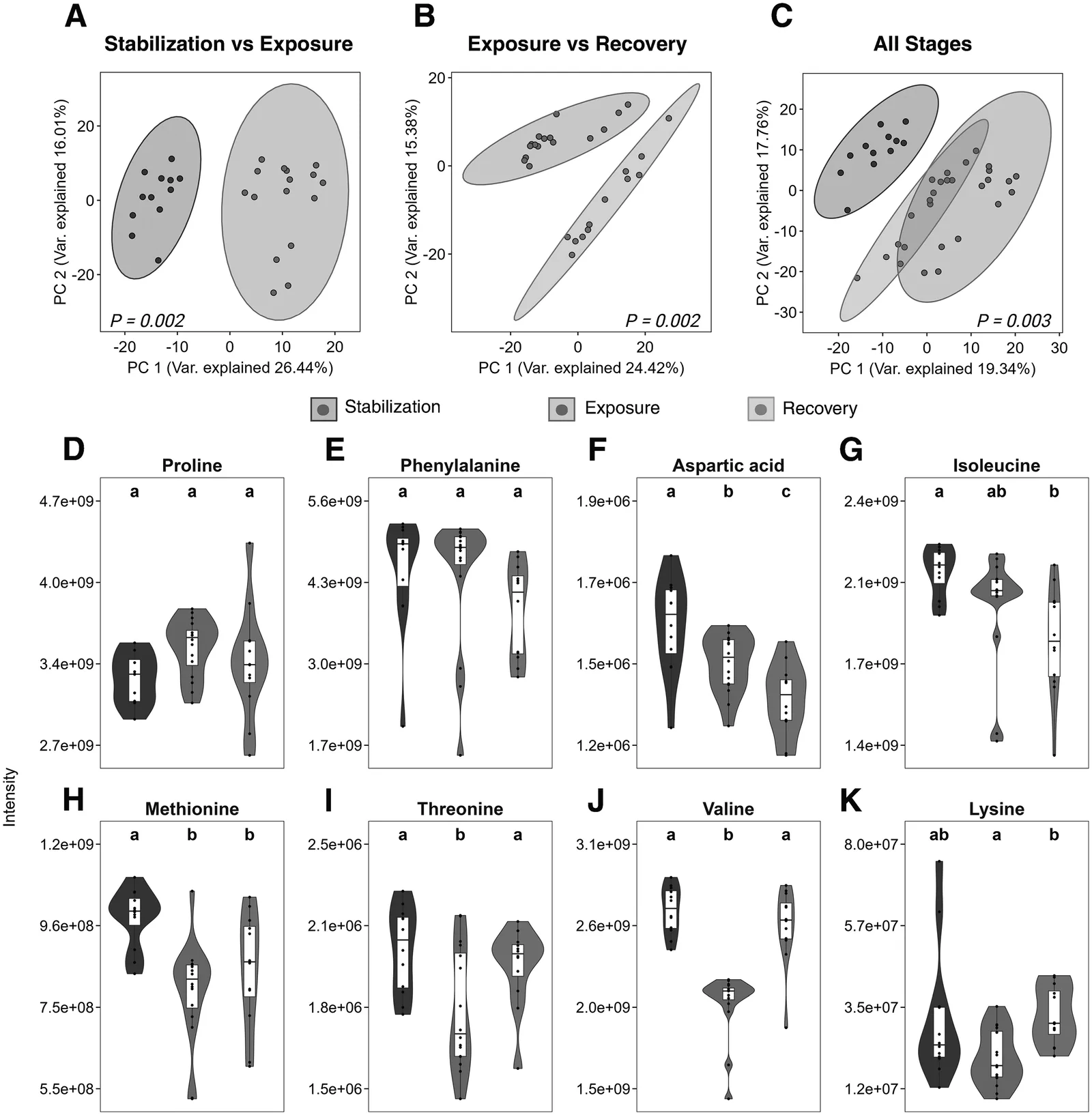
Untargeted metabolomic changes in SIHUMIx following azoxystrobin exposure. PCA of metabolite intensities comparing A) stabilization to exposure, B) exposure to recovery, and C) across all experimental stages; statistical significance determined using PERMANOVA. D-K) Violin plots depicting the distribution and density of 8 amino acid intensities across the different cultivation phases. Letters above each plot indicate significant differences between experimental phases, determined from linear mixed-effects models with bioreactor as a random effect and Tukey-adjusted post-hoc comparisons (*P* < 0.05). Individual points represent measurements from the 4 bioreactors at each phase.

Untargeted metabolomics detected 638 metabolites across the 3 experimental stages. Profile comparisons showed significant shifts between stabilization and exposure (PERMANOVA, *P* = 0.002) (Fig. 6A) and between exposure and recovery (PERMANOVA, *P* = 0.002) (Fig. 6B). Amino acid metabolism emerged as a key target of azoxystrobin exposure during pathway analysis. Of the 20 essential amino acids, 15 were detected, with asparagine, glutamine, glycine, histidine, and leucine absent from the results. Of the detected amino acids, proline and phenylalanine levels remained comparable across the stabilization, recovery, and exposure phases (Fig. 6D and E). Aspartic acid, isoleucine, and methionine levels decreased during exposure, significantly so for aspartic acid and methionine (LMM, *P* = 0.03 and *P* = 0.001, respectively) (Fig. 6F to H). From exposure to recovery, aspartic acid levels continued to decrease significantly (LMM, *P* = 0.04), whereas methionine levels remained similar to the decreased levels detected during exposure. Threonine and valine levels also decreased during the exposure period (LMM, *P* = 0.002 and *P* < 0.001, respectively); however, both amino acids, as well as lysine, increased significantly from exposure to recovery (LMM, *P* < 0.05 for all), returning to levels comparable to stabilization (Fig. 6I to K).

### Defined microbiota colonization selectively persists in larval zebrafish

**Fig. 7.**
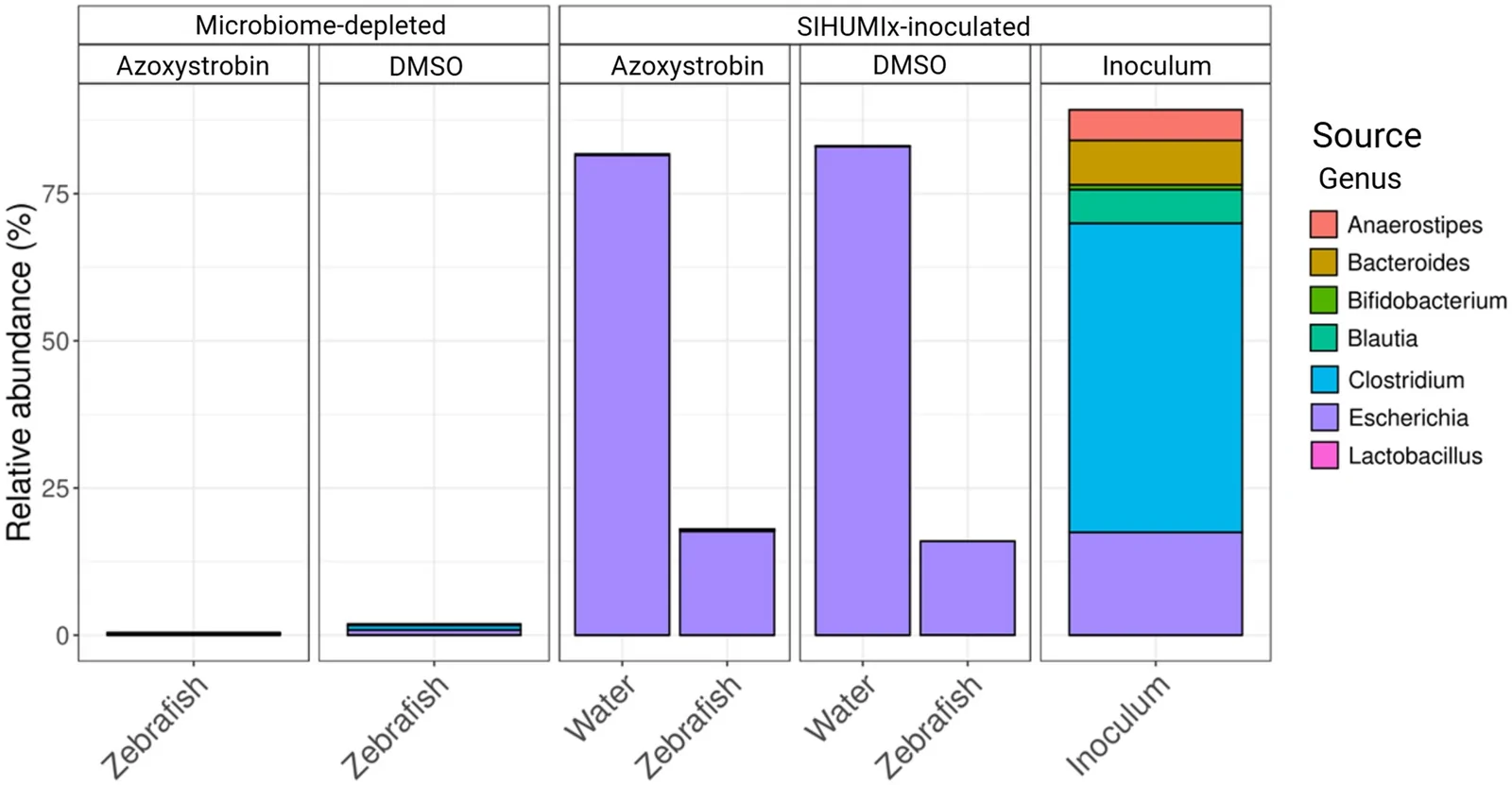
Microbiota characterization of larval zebrafish. Stacked barplots show the relative abundance of SIHUMIx-targeted genera detected in larval zebrafish, media samples (“Water”), and the SIHUMIx inoculum used to generate the SIHUMIx-inoculated zebrafish cohort (prepared as described in the “Bacterial culturing and preparation for larval zebrafish exposure” Section), all analyzed by 16S rRNA gene sequencing. Only taxa corresponding to the 8-member defined microbial consortium are shown (full taxonomic profile in Fig. S6). Data represent pools of 10 larvae per microbiome colonization status and chemical exposure (*n* = 7 to 8), media samples from inoculated flasks per chemical exposure (*n* = 3), and the SIHUMIx inoculum (*n* = 3).

To examine whether azoxystrobin altered neurobehavioral responses in zebrafish larvae in a microbiome-dependent manner, we generated microbiome-depleted larvae and inoculated a subset of these larvae at 1 dpf to form the SIHUMIx-inoculated cohort. 16S rRNA gene sequencing was used to determine whether any bacterial strains from the SIHUMIx persisted in the media and if they were detectable in the larval zebrafish. Microbiome-depleted larvae exhibited minimal detection of SIHUMIx-associated taxa regardless of treatment (Fig. 7). In contrast, SIHUMIx-inoculated larvae were dominated by *E. coli*, with media samples taken from flasks housing the larvae showing high relative abundance of the same genus (Fig. 7). In addition to SIHUMIx-associated taxa, 16S rRNA gene sequencing identified bacterial genera not included in the SIHUMIx inoculum in both microbiome-depleted and SIHUMIx-colonized larvae, including *Bradyrhizobium*, *Brevundimonas, Herbaspirillum*, *Pelomonas*, *Pseudomonas*, *Ralstonia*, *Sphingomonas*, *Variovorax*, and unclassified bacterial taxa (Fig. S6). However, as microbial growth was not present in flask water using traditional microbiological methods, we interpret these reads as reflecting low-level background bacterial DNA contamination rather than viable, actively colonizing microbiota.

### Behavior is modulated by azoxystrobin exposure and microbial status

We used the VAMR new approach method (NAM) to investigate whether azoxystrobin exposure altered neurobehavioral responses in larval zebrafish. Furthermore, we generated larvae with different colonization statuses (microbiome-depleted, SIHUMIx-inoculated) to determine whether observed behavioral effects were potentially microbiota-dependent. Behavioral responses were interpreted relative to the SIHUMIx-inoculated DMSO-exposed vehicle control group, which represents a benchmark for colonized and unexposed conditions.

**Fig. 8.**
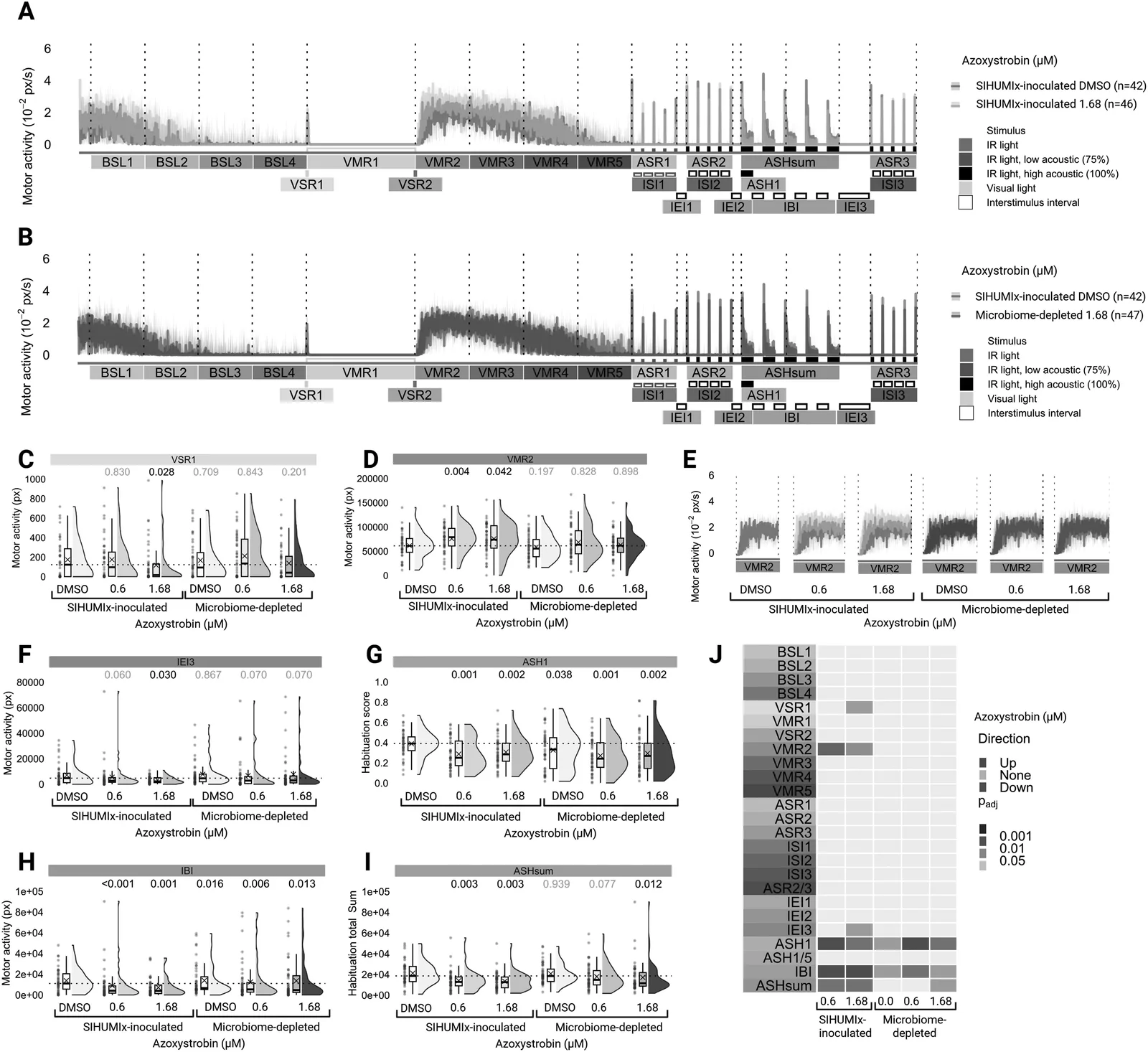
Motor activity of microbiome-depleted and SIHUMIx-inoculated zebrafish larvae exposed to azoxystrobin across various stimulus modalities. A and B) Motor activity (*y-*axis) of SIHUMIx-inoculated zebrafish larvae exposed to 0.4% DMSO (*n* = 42, gray) is compared with A) SIHUMIx-inoculated zebrafish larvae exposed to 1.68 µM azoxystrobin (*n* = 46, yellow) and B) microbiome-depleted zebrafish larvae exposed to 1.68 µM azoxystrobin (*n* = 47, blue). Data are median ± 95% CI. E) A subset of VAMR line plots highlighting motor activity during the visual motor response (VMR2) endpoint. Raincloud plots quantify motor activity and habituation metrics across stimulus modalities. Motor responses are shown under C) illuminated and D) non-illuminated (dark) conditions, F) during inter-endpoint intervals, H) during inter-bout intervals, G) during acoustic startle habituation, and I) as total motor activity across all 5 habituation bouts calculated as the acoustic startle habituation sum (ASHsum). Cohorts from both colonization statuses were exposed to DMSO, 0.6 µM, or 1.68 µM azoxystrobin; statistical significance was assessed relative to the SIHUMIx-inoculated DMSO group. Horizontal dotted lines indicate median motor activity of vehicle-exposed SIHUMIx-inoculated larvae. Numbers above the rainclouds represent Benjamini–Hochberg adjusted *P*-values (gray: *P* ≥ 0.05, black: *P* < 0.05; 2-sample bootstrapping test). J) A heatmap of *P*-values comparing median responses to the vehicle control across all endpoints (*y-*axis) and experimental groups (*x-*axis). Heatmap colors represent *P*-value comparisons to the colonized control group, with gray representing no significant difference, and red and blue indicating increased and reduced motor activity, respectively. Sample sizes: SIHUMIx-inoculated DMSO (*n* = 42), SIHUMIx-inoculated 0.6 µM (*n* = 45), SIHUMIx-inoculated 1.68 µM (*n* = 46), microbiome-depleted DMSO (*n* = 45), microbiome-depleted 0.6 µM (*n* = 47), and microbiome-depleted 1.68 µM (*n* = 47).

During the initial dark acclimation period, larval motor activity across all microbial colonization and exposure conditions peaked approximately 5 min after the plate was transferred to the Zebrabox (BSL1) before returning to baseline levels (BSL2 to BSL4). At the dark-to-light transition (VSR1), we observed a significant decrease (2-sample bootstrap, *P* = 0.028) in startle activity for SIHUMIx-inoculated larvae exposed to 1.68 µM azoxystrobin (Fig. 8C). In the first 5 min after the subsequent light-to-dark transition (VMR2), SIHUMIx-inoculated control larvae exhibited a stereotyped increase in motor activity. In this endpoint, colonized larvae exposed to azoxystrobin exhibited significant hyperactivity compared with colonized controls (2-sample bootstrap, *P* = 0.004 and *P* = 0.042 for 0.6 and 1.68 µM, respectively) (Fig. 8D and E). This effect was not observed in microbiome-depleted cohorts. Activity normalized in later dark-phase endpoints (VMR3 to VMR5) across all groups, with motor activity returning to baseline levels.

Both colonized and microbiome-depleted control larvae exhibited the stereotyped startle responses to acoustic stimuli, with 5 low-intensity stimuli (1 s) eliciting startle responses of lower amplitude (ASR1) compared with the higher amplitude responses (ASR2 and ASR3) evoked by 5 high-intensity acoustic stimuli. Azoxystrobin exposure did not significantly alter these responses in either colonization group relative to the colonized controls. During the intervals between the acoustic stimuli (ISI1 to ISI3), as well as during the recovery periods immediately following ASR1 and ASR2 (IEI1 and IEI2), DMSO-exposed larvae from both colonization cohorts were largely inactive, and azoxystrobin exposure produced no significant deviation from this baseline.

In each of the 5 consecutive bouts of habituation delivered 1 min apart, the SIHUMIx-inoculated control larvae exhibited a progressive reduction in response magnitude, a characteristic feature of habituation. In comparison, during the first habituation bout (ASH1), SIHUMIx-inoculated larvae exposed to azoxystrobin demonstrated an inappropriate acceleration of habituation (2-sample bootstrap, *P* = 0.001 and *P* = 0.002 for 0.6 and 1.68 µM, respectively) (Fig. 8G). Microbiome-depleted larvae exposed to DMSO alone also displayed an inappropriate acceleration of habituation (2-sample bootstrap, *P* = 0.038), with azoxystrobin exposure further exacerbating the observed phenotype in microbiome-depleted larvae (2-sample bootstrap, *P* = 0.001 and *P* = 0.002 for 0.6 and 1.68 µM, respectively) (Fig. 8G). Total motor activity across all 5 habituation bouts (ASHsum) was significantly reduced in azoxystrobin-exposed larvae from both colonization status groups. SIHUMIx-inoculated larvae exhibited significant hypoactivity at both tested concentrations (2-sample bootstrap, *P* = 0.003 and *P* = 0.003 for 0.6 and 1.68 µM, respectively), whereas microbiome-depleted larvae only exhibited a significant reduction at the higher concentration (2-sample bootstrap, *P* = 0.012) (Fig. 8I).

Between habituation bouts (IBI), SIHUMIx-inoculated larvae exposed to azoxystrobin exhibited significantly decreased motor activity compared with colonized controls (2-sample bootstrap, *P* < 0.001 and *P* = 0.001 for 0.6 and 1.68 µM, respectively) (Fig. 8H). Larvae in the microbiome-depleted DMSO group also exhibited less swimming behavior compared with colonized controls (2-sample bootstrap, *P* = 0.016), with azoxystrobin exposure further enhancing the observed hypoactivity in depleted larvae (2-sample bootstrap, *P* = 0.006 and *P* = 0.013 for 0.6 and 1.68 µM, respectively). In the 3-min recovery period following the habituation bouts (IEI3), colonized larvae exposed to 1.68 µM showed a decrease in motor activity compared with the colonized controls (2-sample bootstrap, *P* = 0.030) (Fig. 8D). This effect was not observed in microbiome-depleted larvae.

### Colonization status shapes metabolic profile of larvae following chemical exposure

**Fig. 9.**
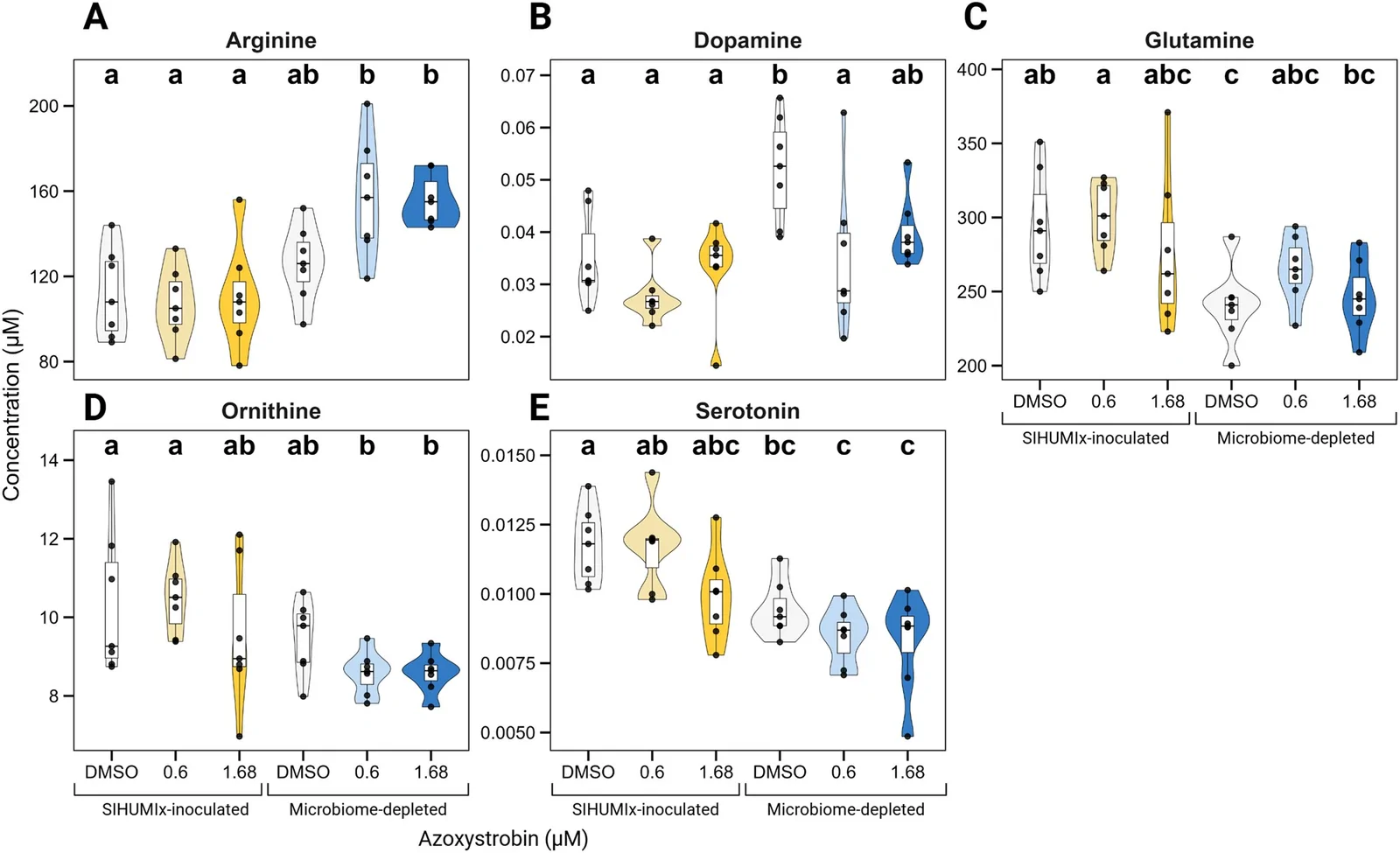
Metabolite abundances across microbial colonization statuses and azoxystrobin exposure conditions. A to E) Concentrations of 5 significant metabolites in larval zebrafish collected at 5 dpf. Statistical analysis was conducted according to data distribution: Normally distributed metabolites (arginine, serotonin, glutamine, and dopamine) were analyzed using a 2-way ANOVA followed by Tukey’s HSD post-hoc test (*P* < 0.05), whereas non-normally distributed metabolites (ornithine) were assessed using the KW test followed by Dunn’s post-hoc test with Benjamini–Hochberg correction for multiple testing (*P* < 0.05). Different letters above the violin plots indicate significant pairwise differences. Each data point represents a pool of 10 larvae.

To investigate the metabolic impact of microbial colonization and azoxystrobin exposure, we quantified 28 metabolites in larval zebrafish, including amino acids, biogenic amines, and SCFAs. Of the metabolites, 22 were normally distributed and analyzed using a 2-way ANOVA, whereas 6 were non-normally distributed and analyzed with the KW test. Corresponding post-hoc comparisons revealed significant differences across microbial and exposure conditions for arginine, serotonin, glutamine, dopamine, and ornithine (Fig. 9A to E). Arginine levels increased progressively across conditions, with the highest concentrations observed in microbiome-depleted larvae exposed to azoxystrobin (Fig. 9A). Ornithine levels, by contrast, decreased across conditions, with the lowest levels in depleted-exposed groups (Fig. 9D). Dopamine was elevated in microbiome-depleted DMSO-treated larvae but was reduced to levels comparable to colonized counterparts following exposure to azoxystrobin (Fig. 9B). Serotonin and glutamine levels were both lower in microbiome-depleted DMSO-treated larvae compared with colonized controls (Fig. 9C and E). In colonized larvae, serotonin levels decreased with increasing azoxystrobin exposure (Fig. 9E). In contrast, serotonin levels in microbiome-depleted larvae remained consistently low, regardless of chemical exposure, indicating a microbiota-dependent reduction with limited additional impact from the chemical (Fig. 9E).

To contextualize the observed shifts in metabolite abundances, significant compounds were subjected to zebrafish-specific KEGG pathway enrichment analysis. This identified several metabolic pathways potentially influenced by colonization status and azoxystrobin exposure, including arginine biosynthesis, arginine and proline metabolism, tryptophan metabolism, alanine, aspartate, and glutamate metabolism, and tyrosine metabolism (Fig. S8).

## Discussion

SIHUMIx is a powerful tool to structurally and functionally investigate the impact of xenobiotics with a human-relevant ex vivo microbiota (Castañeda-Monsalve et al. 2024, 2025; Fischer et al. 2024; Haange et al. 2024). A previous SIHUMIx screening of 90 xenobiotics identified azoxystrobin as a potent metabolic disruptor, though the 24 h exposure duration potentially limited detection of relevant chemical–microbiome interactions (Castañeda-Monsalve et al. 2024). To address this gap, we used the SIHUMIx model to evaluate the effects of prolonged azoxystrobin exposure followed by a recovery phase to better understand whether exposure elicits persistent effects on protein biomass-based community structure and metabolic function. Another constraint of the SIHUMIx system is that it only assesses the direct effects of compounds on the microbiota, without capturing host-mediated influences or the role of host-produced metabolites. To address this, we combined a controlled microbiota system with a larval zebrafish model to interrogate whether azoxystrobin exposure causes microbiome-dependent effects at the host level.

Azoxystrobin exposure resulted in pronounced alterations in proteome-weighted SIHUMIx community structure, with significant changes detected in relative protein abundance for 7 out of 8 constituent species. The most notable effect was an increase in *E. coli* (Pseudomonadota, formerly Proteobacteria) abundance, accompanied by decreases in multiple Bacillota species (formerly Firmicutes: *C. butyricum*, *B. producta*, *A. caccae*, *L. plantarum*) and *B. longum* (Actinomycetota, formerly Actinobacteria). Comparable phylum-level shifts have been observed in both *Enchytraeus crypticus* (Oligochaeta) and mice, where azoxystrobin exposure drove Pseudomonadota enrichment and Bacillota depletion (Zhang et al. 2019; Meng et al. 2023). This cross-species convergence suggests that azoxystrobin may broadly disrupt gut microbial balance. In microbiome–host systems, community structure often reverts to its initial constellation following disruption by chemical exposure (Narrowe et al. 2015; Hu et al. 2016; Liu et al. 2020; Ouyang et al. 2021; Quintanilla-Mena et al. 2021; Zhang et al. 2022), a process termed the “rebound” phenomenon. Importantly, rebound has also been demonstrated within SIHUMIx after both a PFAS mixture exposure (Haange et al. 2024) and pulse and press disturbances (Schäpe et al. 2019; Krause et al. 2020). In contrast to these findings, azoxystrobin exposure led to a persistently altered SIHUMIx state even after chemical removal. Persistently altered community structures are well recognized as markers of certain host genetic backgrounds and disease contexts (Blekhman et al. 2015; Miyake et al. 2015; Pierce et al. 2024), indicating that lasting SIHUMIx shifts triggered by azoxystrobin exposure may reflect an altered microbiota composition with potential consequences for host health.

To explore whether the observed community-level shifts translated to a vertebrate host, we associated microbiome-depleted zebrafish larvae with SIHUMIx. Similar to the SIHUMIx results, *E. coli* dominated the zebrafish microbiota following azoxystrobin exposure. However, *E. coli* was the only member of the SIHUMIx consortium detected at 5 dpf, regardless of azoxystrobin exposure, preventing evaluation of community-level changes. This outcome likely reflects the competitive dominance of *E. coli* combined with selective pressures in the oxygenated early zebrafish gut (Rawls et al. 2004) and is consistent with prior reports that only a subset of human gut microbes persists in zebrafish larvae (Toh et al. 2013; Valenzuela et al. 2018). When examining the full taxonomic profile of genera detected across experimental conditions, 16S rRNA gene sequencing revealed additional bacterial genera in microbiome-depleted larvae despite negative culture-based sterility testing. This likely reflects the higher sensitivity of sequencing-based approaches relative to conventional culture assays and has been reported previously in germ-free-derived zebrafish models (Weitekamp et al. 2021). The taxa detected were commonly reported background or reagent-associated genera (Tanner et al. 1998; Grahn et al. 2003; Barton et al. 2006; Laurence et al. 2014; Salter et al. 2014) and exhibited similar compositional patterns across microbiome-depleted and SIHUMIx-colonized groups. However, as microbial growth was not present in flask water using traditional microbiological methods, we interpret these reads as reflecting low-biomass background DNA signal rather than an established, viable microbial community. Overall, although SIHUMIx provides mechanistic insight into microbial shifts, early life-stage zebrafish colonization is constrained by microbial traits, colonization timing, and gut physiology. Future studies should consider using older zebrafish, where human-relevant anaerobic microbes may have a higher likelihood of colonizing the experimental host, and incorporating baseline sampling of unexposed larvae paired with gut-dissection-based approaches to clarify the relative contributions of intestinal versus extra-intestinal microbial communities.

To expand our investigation of azoxystrobin exposure beyond structural changes, we quantified SCFAs and the intermediate molecule lactate across cultivation phases. As the major end products of bacterial fermentation of dietary fibers, resistant starch, proteins, and peptides, SCFAs play key roles in host physiology and immune regulation (Blaak et al. 2020; Hirmas et al. 2022). In SIHUMIx, azoxystrobin exposure significantly reduced lactate and butyrate during the exposure phase, with both remaining suppressed in the recovery period. These results align with, and extend, previous observations in SIHUMIx (Castañeda-Monsalve et al. 2024) where butyrate levels were also affected after 24 h exposure, and in mice (Meng et al. 2023), where azoxystrobin exposure reduced lactate and acetate levels. Mechanistically, azoxystrobin exposure significantly suppressed key metabolic enzymes, including pyruvate kinase, lactate dehydrogenase, acetyl-CoA C-acetyltransferase, and butyryl-CoA dehydrogenase, consistent with reduced lactate and butyrate. These results suggest that azoxystrobin impairs microbial fermentation pathways in SIHUMIx, with effects on both intermediate and terminal steps of SCFA production. Decreases in butyrate may be further compounded by the reduction of *C. butyricum*, a key butyrate producer in the SIHUMIx strains (Becker et al. 2011). Together, these findings highlight that azoxystrobin may impact microbial fermentation through both enzyme inhibition and community compositional shifts, with potential consequences for host energy metabolism and gut health.

To better contextualize the observed shifts in metabolite profiles, we integrated metaproteomic data with KEGG pathway mapping. This revealed notable functional alterations between SIHUMIx stabilization and exposure stages, with changes in 23 of 84 identified pathways. Pathways linked to cofactor and vitamin biosynthesis, nutrient acquisition, and detoxification were upregulated, reflecting a coordinated metabolic reprogramming of the microbial community under azoxystrobin exposure. Concurrent activation of chemotaxis and transport systems suggests that bacteria invested resources not only in maintaining core metabolism but also in actively adapting to the environment. Interestingly, azoxystrobin exposure in batch mode (24 h) led to the downregulation of protein export and bacterial secretion pathways (Castañeda-Monsalve et al. 2024), suggesting early stress responses may involve temporarily suppressing energy-demanding functions, whereas extended exposure may promote coordinated metabolic and adaptive responses. In addition, longer-term exposure led to the downregulation of central metabolic processes, including carbohydrate fermentation, amino acid turnover, and redox pathways, consistent with the observed decrease in SCFA levels. This pattern also aligns with literature showing that mice exposed to azoxystrobin exhibited altered amino acid and sulfur metabolism (Meng et al. 2023), supporting the relevance of these microbial responses across hosts.

Although our findings in SIHUMIx demonstrate structural and metabolic disruption following azoxystrobin exposure, they do not reveal whether such changes translate into host-level phenotypes. We therefore used larval zebrafish to test whether azoxystrobin exposure elicits effects on host behavior. Assessing locomotor behavior in zebrafish larvae provides a quick means to detect neurobehavioral effects of chemical exposure, as alterations in their stereotyped responses often reflect disturbances in the neural circuits governing these behaviors (Noyes et al. 2015; Bruni et al. 2016; Gaballah et al. 2020; Jarema et al. 2022). We subjected larval zebrafish to the VAMR NAM, which quantifies a range of visual motor responses, visual and acoustic startle responses, habituation learning, and memory retention (Herold et al. 2025; Leuthold et al. 2025). By utilizing zebrafish with different colonization statuses, we can also assess whether observed changes in neurobehavioral profiles are microbiome-dependent (Phelps et al. 2017; Catron et al. 2019; Stagaman et al. 2024). We observed azoxystrobin-dependent effects on non-associative habituation learning that were independent of colonization status. This indicates that some behavioral endpoints may be influenced directly by azoxystrobin. Previous docking analyses (Yang et al. 2021) suggest that azoxystrobin has higher predicted interaction potency with multiple endocrine-related targets in comparison to other strobilurin fungicides, consistent with reports of endocrine disruption in zebrafish following azoxystrobin exposure (Cao et al. 2016). Moreover, environmental endocrine disruptors have been shown to alter learning and habituation endpoints across species (Xu et al. 2010; Mersha et al. 2015). Taken together, these observations support the possibility that receptor-mediated or endocrine-disrupting host-level mechanisms may contribute to the microbiome-independent behavioral effects we observed.

In addition to effects on habituation learning, we observed azoxystrobin-induced dark-phase hyperactivity. Similar dark-phase hyperactivity has been reported previously in azoxystrobin-exposed zebrafish larvae subjected to a dark photokinesis behavioral assay (Yang et al. 2021). Importantly, in our study, the dark-phase hyperactivity phenotype was only observed in SIHUMIx-inoculated larvae. In contrast, microbiome-depleted larvae exposed to the same concentrations of azoxystrobin exhibited control-like behavior. This finding adds to a limited but growing body of work demonstrating a microbiota-dependent modulation of chemical effects on zebrafish development (Gugescu et al. 2025) and behavior (Catron et al. 2019; Stagaman et al. 2024). More broadly, it supports the concept that microbiota mediate chemical toxicity (Haiser et al. 2013; Coryell et al. 2018; Catron et al. 2019; Brinkmann et al. 2020) and demonstrates that chemical–microbial interactions can manifest at the level of host neurobehavioral responses.

To explore potential mediators of the microbiota-dependent dark-phase hyperactivity, we quantified neurotransmitter levels. SIHUMIx-inoculated control larvae had higher baseline serotonin than microbiome-depleted larvae, consistent with evidence in mice showing that the gut microbiota influences host serotonin levels (Yano et al. 2015). In colonized larvae, azoxystrobin exposure reduced serotonin, bringing levels closer to those of depleted controls. However, serotonin in colonized, exposed larvae still remained higher than in depleted fish at both tested concentrations, significantly so at the lower concentration. Therefore, the observed dark-phase hyperactivity that was specific to exposed colonized larvae suggests that microbiota-dependent serotonergic activity may shape host responses to azoxystrobin. Future work is needed to determine whether serotonin levels and the observed hyperactivity phenotype are independent phenomena or causally related.

A 2018 report by the National Academies of Sciences, Engineering, and Medicine reviewed studies on microbiota interactions with environmental chemicals and highlighted several gaps that complicate the integration of these interactions into risk assessment frameworks (National Academies of Sciences, Engineering, and Medicine 2018). Among these uncertainties is a need for standardized models, functional evaluation beyond community composition, and a demonstration of causality. Subsequent reports from the European Food Safety Authority (Ampatzoglou et al. 2022; Moreno et al. 2024) and the Food and Agriculture Organization emphasize many of the same challenges (FAO 2023), highlighting the continued relevance of these issues. This azoxystrobin study addresses several of these priorities. Using the defined SIHUMIx consortium, we provide a reproducible platform to assess azoxystrobin-induced shifts in microbial community structure based on protein biomass. Significant community changes were observed at 10% of the ADI and persisted into the recovery phase, indicating microbial community sensitivity at sub-regulatory exposure levels. Addressing functionality, metaproteomic and metabolite analyses revealed reduced SCFA production and perturbation of key metabolic pathways, suggesting functional disruptions with potential implications for host physiology. To explore causality, we compared the behavior profiles of microbiome-depleted and SIHUMIx-inoculated zebrafish larvae in the VAMR NAM and found that azoxystrobin exposure altered behavior in a microbiome-dependent manner. This integrative ex vivo-to-in vivo approach demonstrates microbiota mediation of chemical responses in a 3R-compliant manner and while addressing critical issues of standardization, functional characterization, and mechanistic understanding. Collectively, these results reinforce the concept that the microbiota is a key determinant of how environmental chemicals influence health and underscore the need for microbiota-informed risk assessment.

## Supplementary Material

kfag022_Supplementary_Data

## Data Availability

The mass spectrometry proteomics data have been deposited to the ProteomeXchange Consortium via the PRIDE (Perez-Riverol et al. 2022) partner repository with the dataset identifier PXD070601. 16S rRNA sequencing data are deposited in the NCBI Sequence Read Archive (https://www.ncbi.nlm.nih.gov/sra/) under BioProject accession PRJNA1364743. Underlying code and data used to generate figures are available at https://doi.org/10.5281/zenodo.18433571 (Wray et al. 2026).
