# Supplementary material for: Integration of human microbiota (SIHUMIx) and zebrafish models reveals microbiome-mediated host responses to azoxystrobin
Source: Toxicol Sci. 2026 Mar 7;209(4):kfag022. doi: 10.1093/toxsci/kfag022 (PMC13105170; doi:10.1093/toxsci/kfag022)
Supplement: kfag022_Supplementary_Data [file kfag022_supplementary_data.zip › 06-Apr-2026_020836_20260203_supp_tables_and_figures.pdf]

Supplemental Material

Integration of human microbiota (SIHUMIx) and zebrafish models reveals microbiome-mediated host responses to azoxystrobin

Chloe Wray[1,3*], Victor Castañeda-Monsalve[2*,5+], Beatrice Engelmann[2], Ulrike E. Rolle-Kampczyk[2], Nicole Schweiger[1], Sebastian Gutsfeld[1], Debjyoti Ghosh[2], Siraz Kader[1], Charles R. Tyler[3], Nico Jehmlich[2#], Tamara Tal[1,4#]

[1]Department of Ecotoxicology, Chemicals in the Environment Research Section, Helmholtz-Centre for Environmental Research – UFZ, Leipzig, Germany

[2]Department of Molecular Toxicology, Chemicals in the Environment Research Section, Helmholtz-Centre for Environmental Research – UFZ, Leipzig, Germany

[3]University of Exeter, Biosciences, Geoffrey Pope Building, Stocker Road, Exeter, Devon EX4 4QD, UK

[4]Medical Faculty, University Leipzig, Leipzig, Germany

[5]Proteomics Research Infrastructure, Faculty of Health and Medical Sciences, University of Copenhagen, Denmark

[*]Indicates equal contribution

[+]Indicates current affiliation

[#]Corresponding Authors: Helmholtz Center for Environmental Research – UFZ, Permoserstrasse 15, 04318 Leipzig, Germany. Email: nico.jehmlich@ufz.de; tamara.tal@ufz.de

Supplemental Table

Table S1: Complex intestinal medium (CIM) composition

Ingredient	Quantity [g/L]	Supplier
Arabinogalactan (larch wood)	2	Sigma-Aldrich
Bile Acids sodium salt	0.5	Sigma-Aldrich
Calcium chloride x 2 H_2O	0.01	Merck
Casein peptone (pancreatic)	4.3	Roth
Di-Potassium hydrogen phosphate	0.04	Roth
Hemin (bovine)	0.005	Sigma-Aldrich
Inulin	1	Serva
L-cysteine hydrochloride	0.5	Biochemica
Magnesium sulfate	0.01	Roth
Menadione	0.001	Sigma-Aldrich
Mucin (porcine gastric Type II)	4	Sigma-Aldrich
Pectin, citrus peel	2	Sigma-Aldrich
Potassium di-hydrogen phosphate	0.04	Roth
Sodium chloride	0.72	Roth
Sodium hydrogen carbonate	2	Roth
Starch, wheat	5	Roth
Xylo-oligosaccharide (corn)	2	Roth
Yeast extract	2	Chemsolut

Supplemental Figures

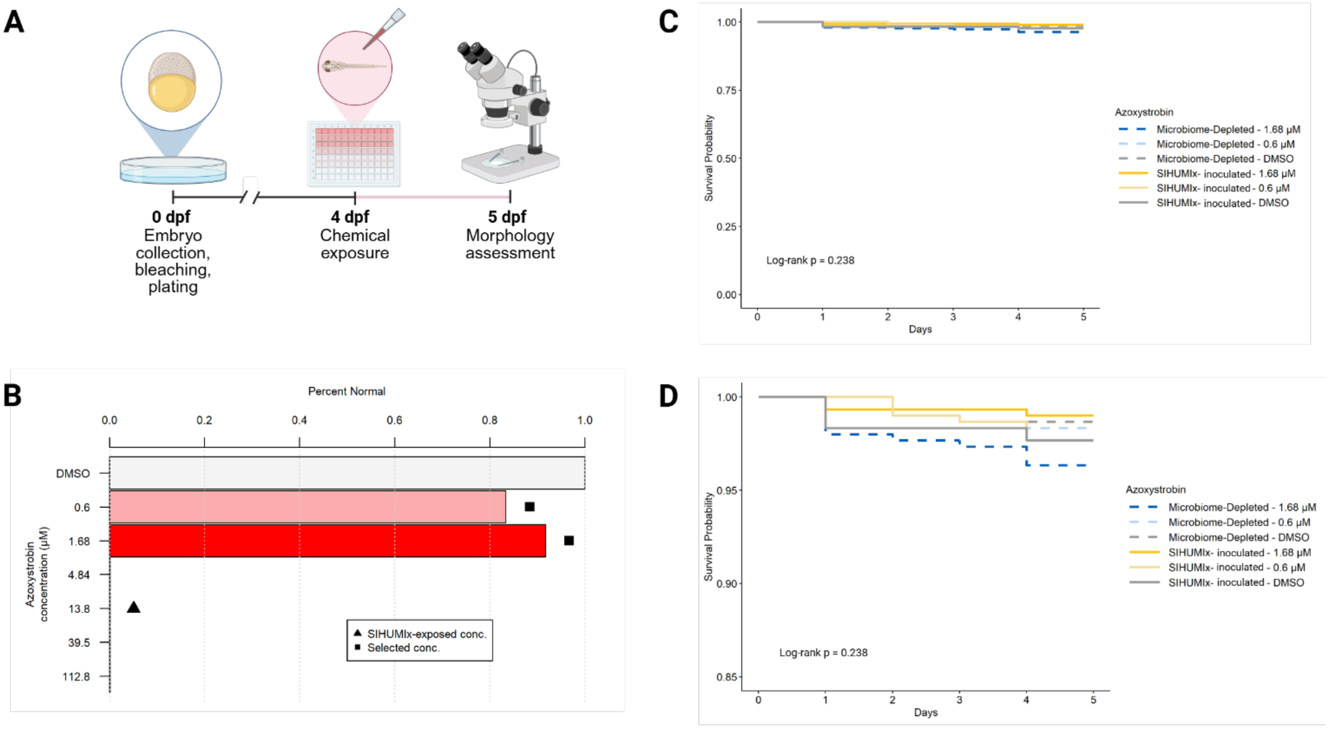

Figure S1. Range-finding exposure paradigm and survival across the experiment. (A) Exposure paradigm for range-finding experiment. Larvae were collected, bleached, and plated at 0 dpf. Larvae were exposed to six concentrations of azoxystrobin (112.8 µM, 39.5 µM, 13.8 µM, 4.84 µM, 1.68 µM, and 0.6 µM) in semi-log spacing. (B) Percent normal zebrafish across tested concentrations. At the highest four concentrations, (112.8 µM, 39.5 µM, 13.8 µM, 4.84 µM), all larvae died. At the lowest two concentrations all larvae survived and had a rate of >0.8 normal morphology. A square to the right of a bar denotes a concentration that was utilized in future experiments, and the triangle represents the concentration used in *ex vivo* SIHUMIx experiments (10% ADI). (C-D) Kaplan-Meier plots showing survival over the course of the zebrafish experiments with different microbiome cohorts. Microbiome status and exposure had no significant effect (P = 0.238) on the survival probability of the larvae. Created in BioRender. Wray, C. (2026) https://BioRender.com/angl3ys

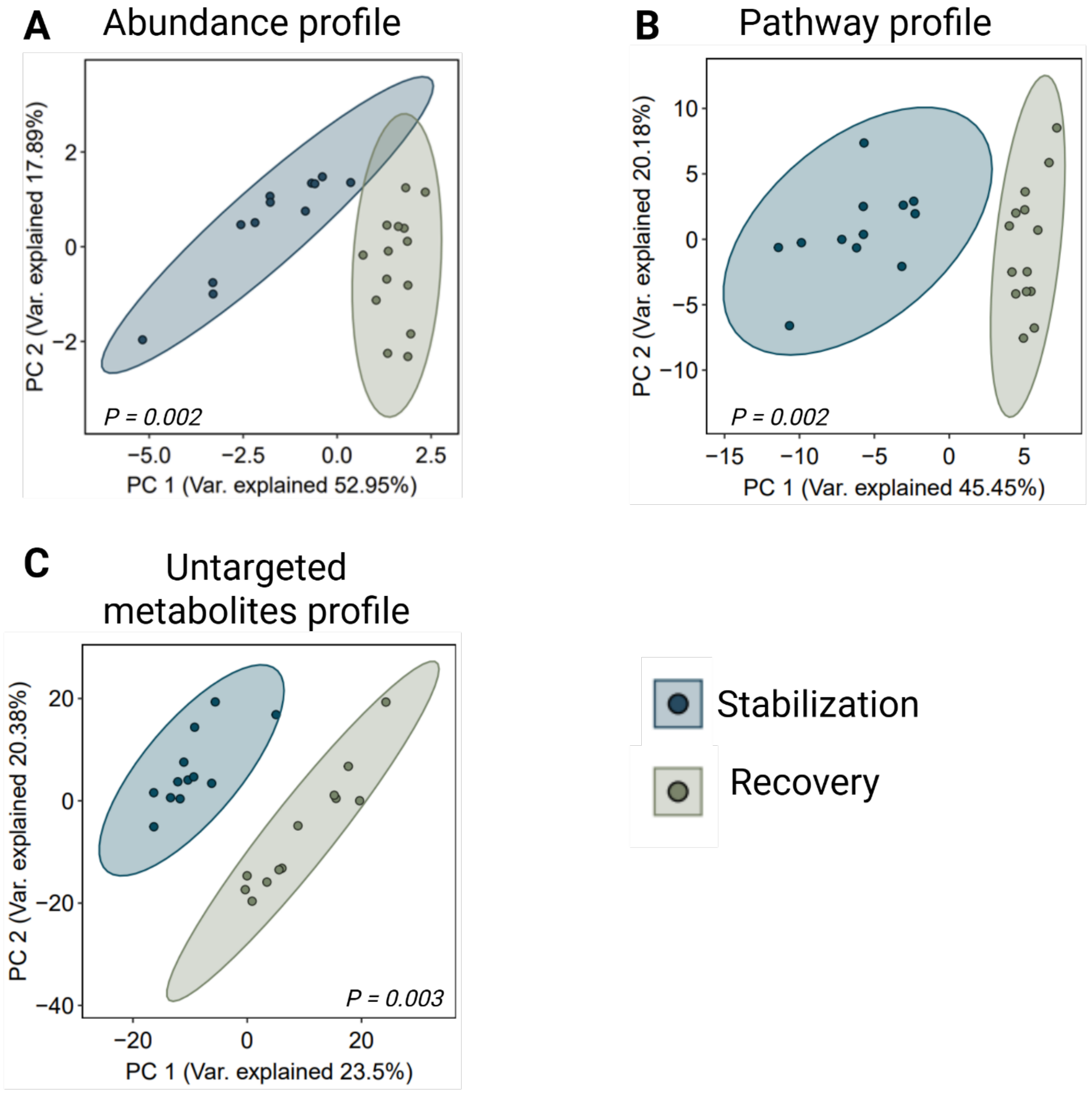

Figure S2. Comparison of stabilization and recovery stages using principal component analysis. (A) Species composition comparisons, (B) metabolic pathway profiles, and (C) the profile of untargeted metabolites. Group separation was tested for significance with pairwise PERMANOVA.

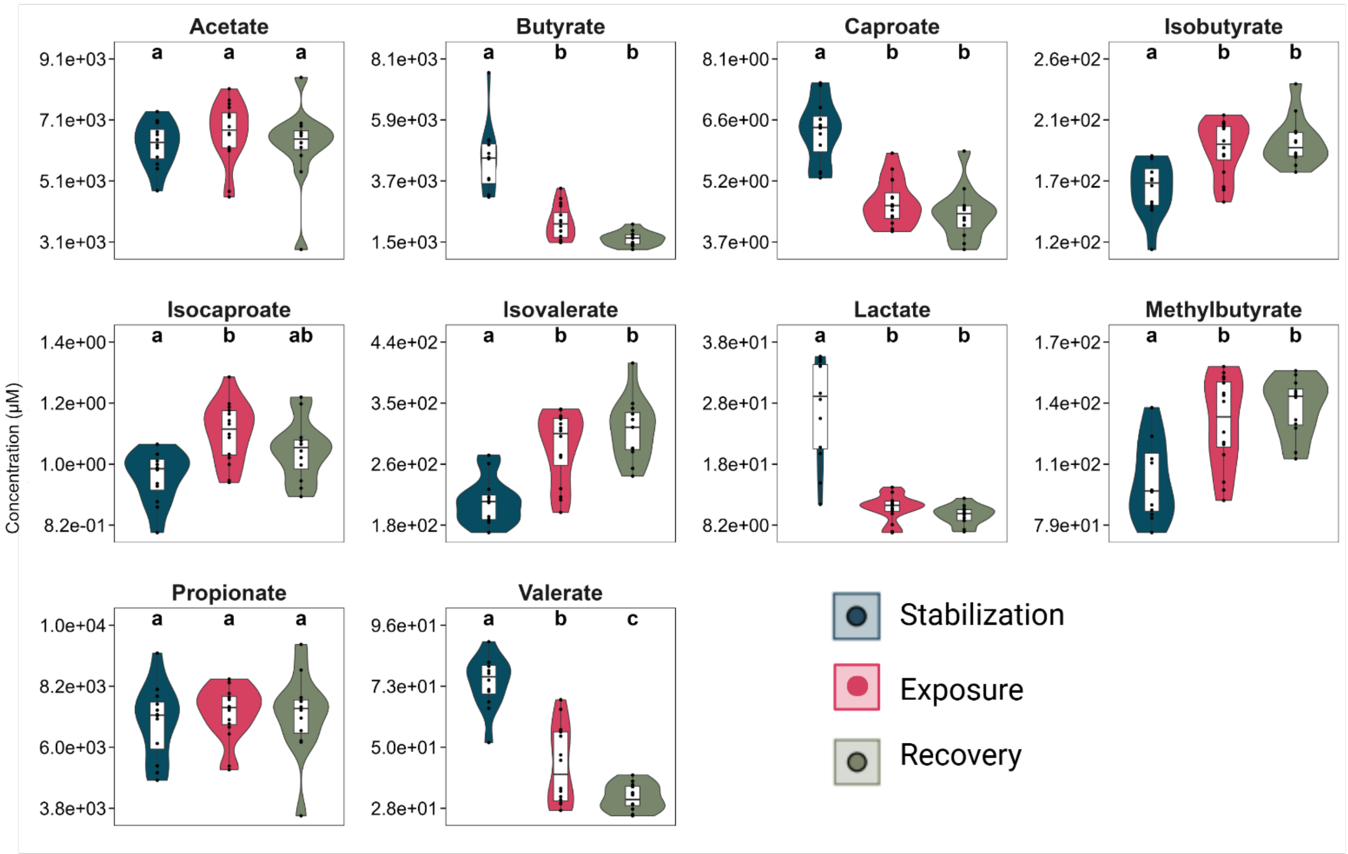

Figure S3. SCFA concentration across experimental stages. The levels of ten short-chain fatty acids (SCFAs) in SIHUMIx supernatants were quantified throughout the different phases of the experiment (Stabilization, Exposure, and Recovery). Letters above each violin indicate significant differences between experimental phases, determined from linear mixed-effects models with bioreactor as a random effect and Tukey-adjusted post-hoc comparisons ($P < 0.05$). Individual points represent measurements from the four bioreactors at each phase.

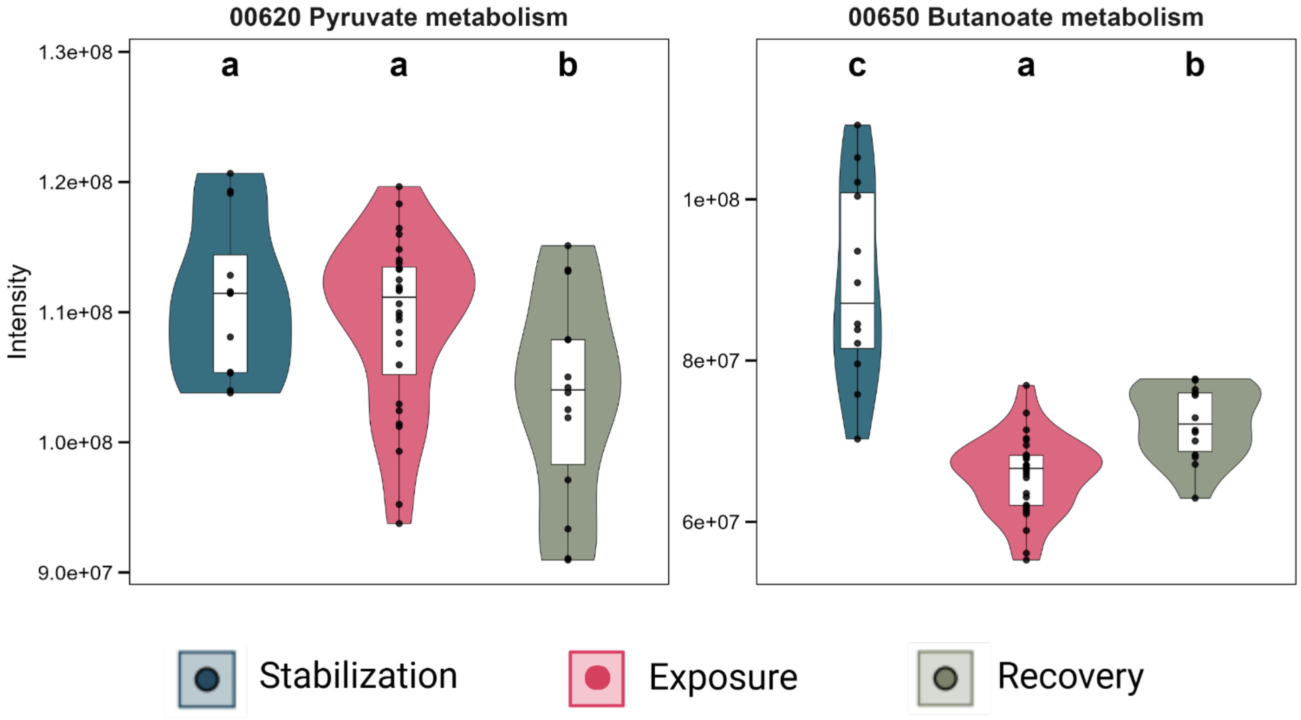

Figure S4. Intensity of pyruvate and butanoate (butyrate) metabolism. The relative abundance of both pathways was assessed before, during, and after exposure to azoxystrobin. Letters above each violin indicate significant differences between experimental phases, determined from linear mixed-effects models with bioreactor as a random effect and Tukey-adjusted post-hoc comparisons ($P < 0.05$). Each point represents an individual sample collected from the four bioreactors across cultivation phases (Stabilization, Exposure, Recovery).

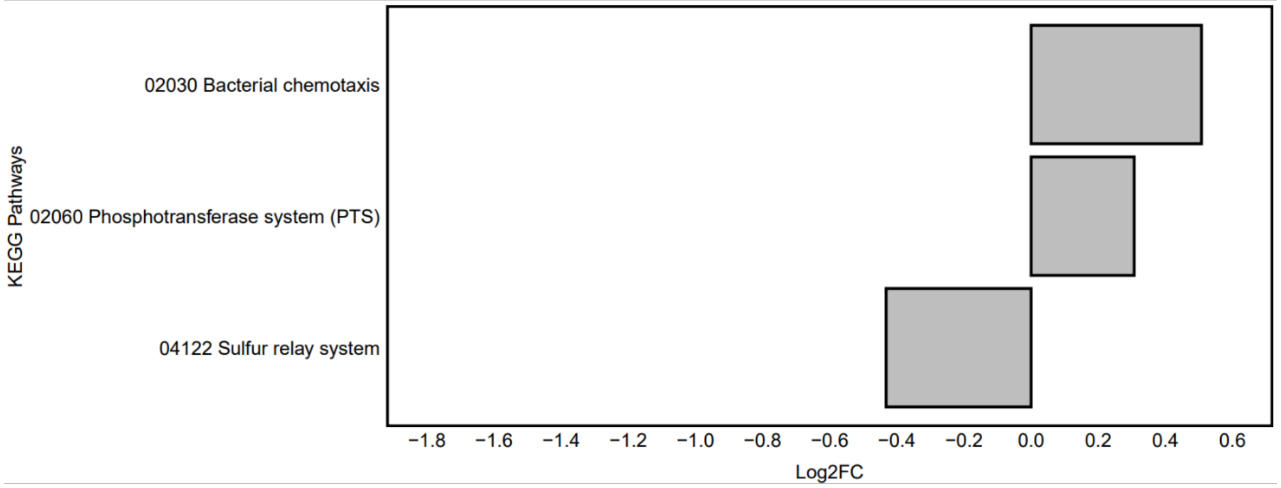

Figure S5. Identification of relevant pathways during the recovery stage. Pathways showing differences between the exposure and recovery phases were identified. Only pathways meeting a Log2 fold-change threshold of ± 0.175 and reaching statistical significance by the Kruskal-Wallis test ($P < 0.05$) are shown.

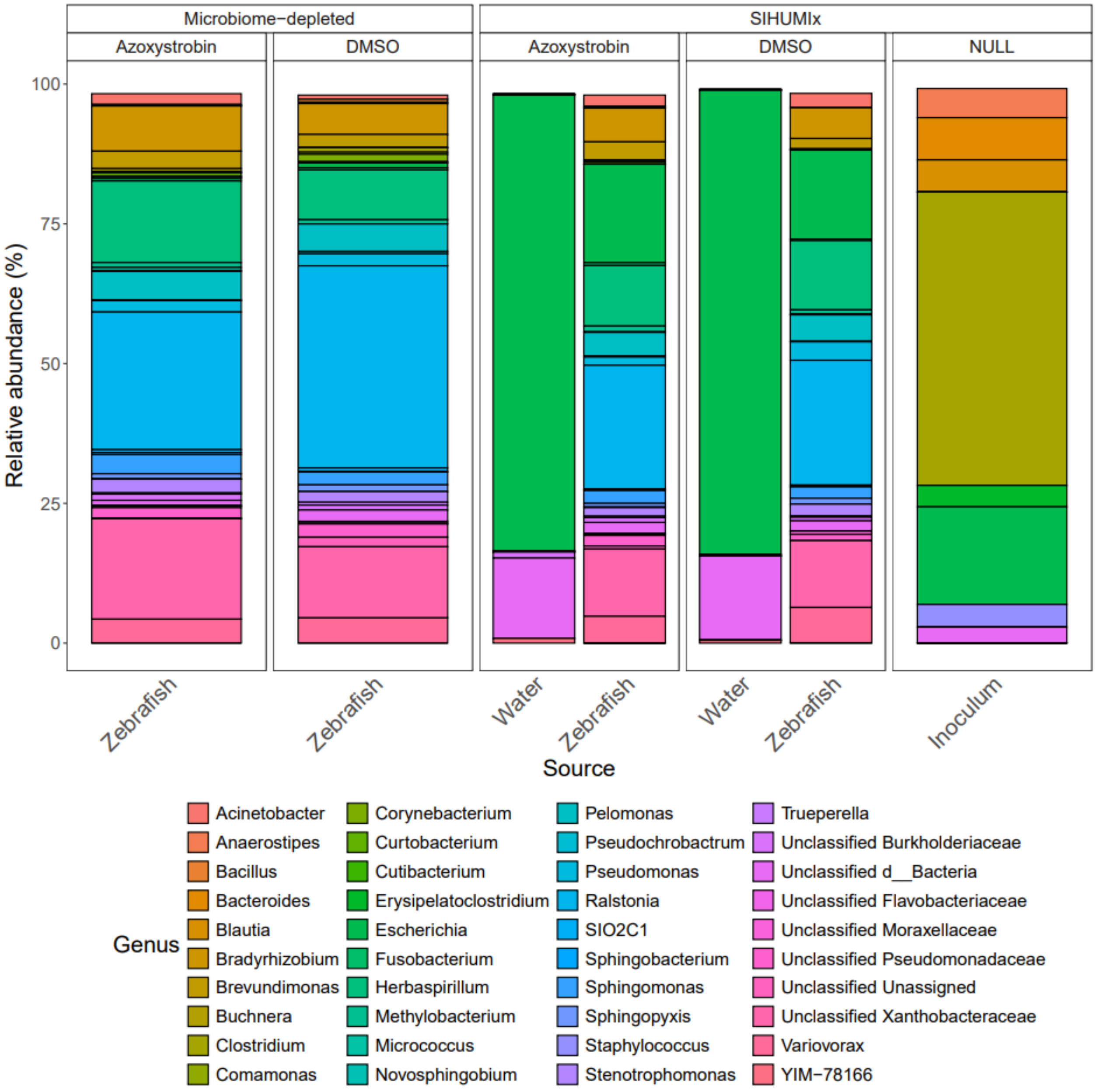

Figure S6. Full taxonomic profile from 16S rRNA sequencing. Stacked bar plots showing the relative abundance of bacterial taxa in microbiome-depleted and SIHUMIx-inoculated larvae under control (DMSO) or azoxystrobin treatment, along with that of media samples ("Water") collected from flasks of inoculated larvae and the SIHUMIx inoculum, including taxa present in at least 10% of samples within each experimental group and that contributed at least 1% relative abundance in at least one individual sample. Each bar represents a single condition with genera color-coded according to assigned taxonomic classification.

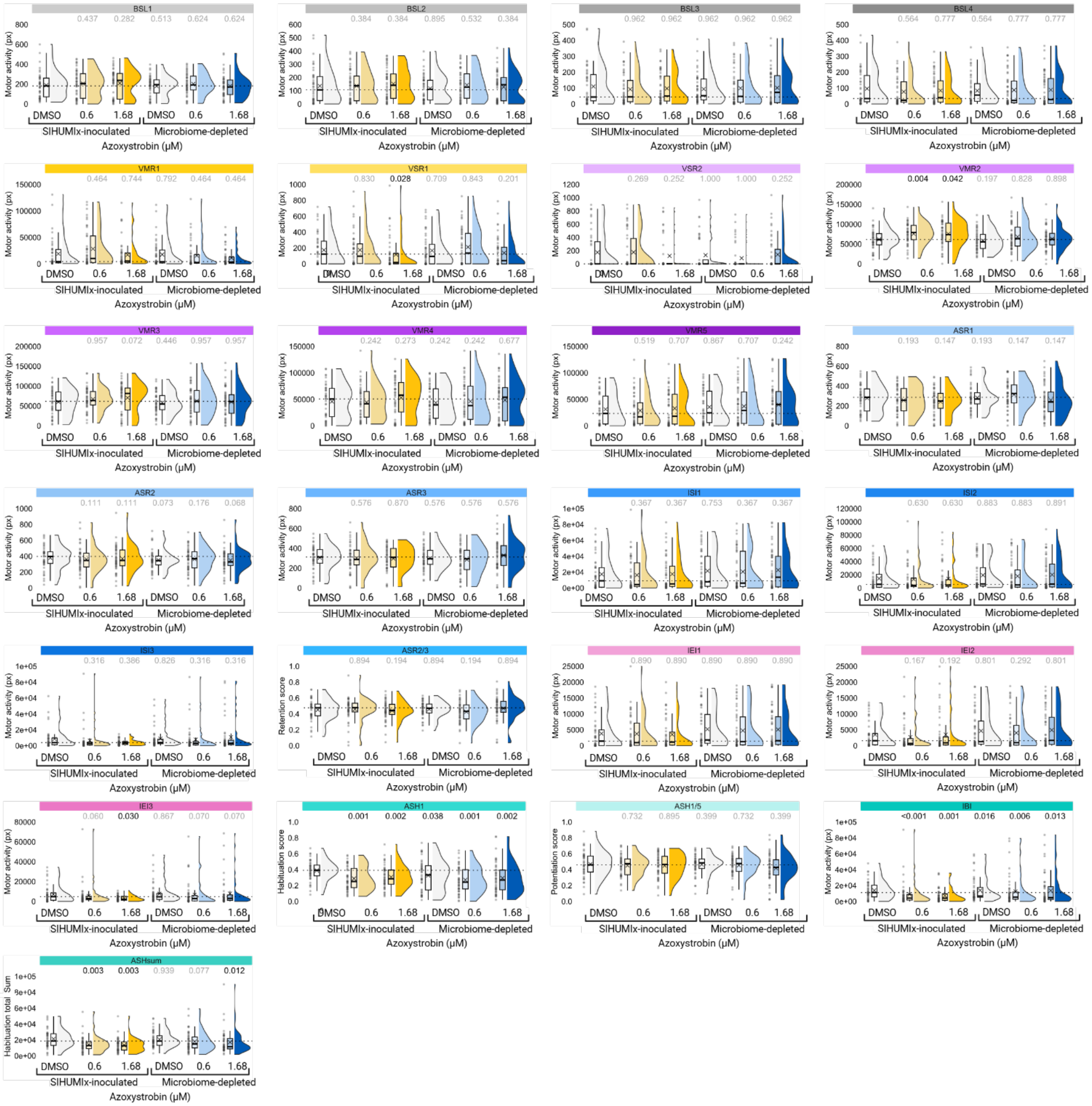

Figure S7. Raincloud plots quantifying motor activity and habituation metrics across all VAMR endpoints. Motor responses are shown during baseline activity (BSL1-BSL4), visual startle responses (VSR1, VSR2), visual motor responses (VMR1-VMR5), acoustic startle responses (ASR1-ASR3), acoustic interstimulus intervals (ISI1-ISI3), inter-acoustic endpoint intervals (IEI1-IEI3), acoustic startle habituation (ASH1), potentiation of habituation (ASH1/5), acoustic habituation sum (ASHsum), inter-bout interval (IBI), and memory retention (ASR2/3). Cohorts from both microbiome statuses were exposed to DMSO, 0.6 µM, or 1.68 µM azoxystrobin; statistical significance was assessed relative to the SIHUMIx-inoculated DMSO group. Horizontal dotted lines indicate median motor activity of vehicle-exposed SIHUMIx-inoculated larvae. Numbers above the rainclouds represent adjusted p-values (gray: $P \geq 0.05$, black: $P < 0.05$; two-sample bootstrapping test).

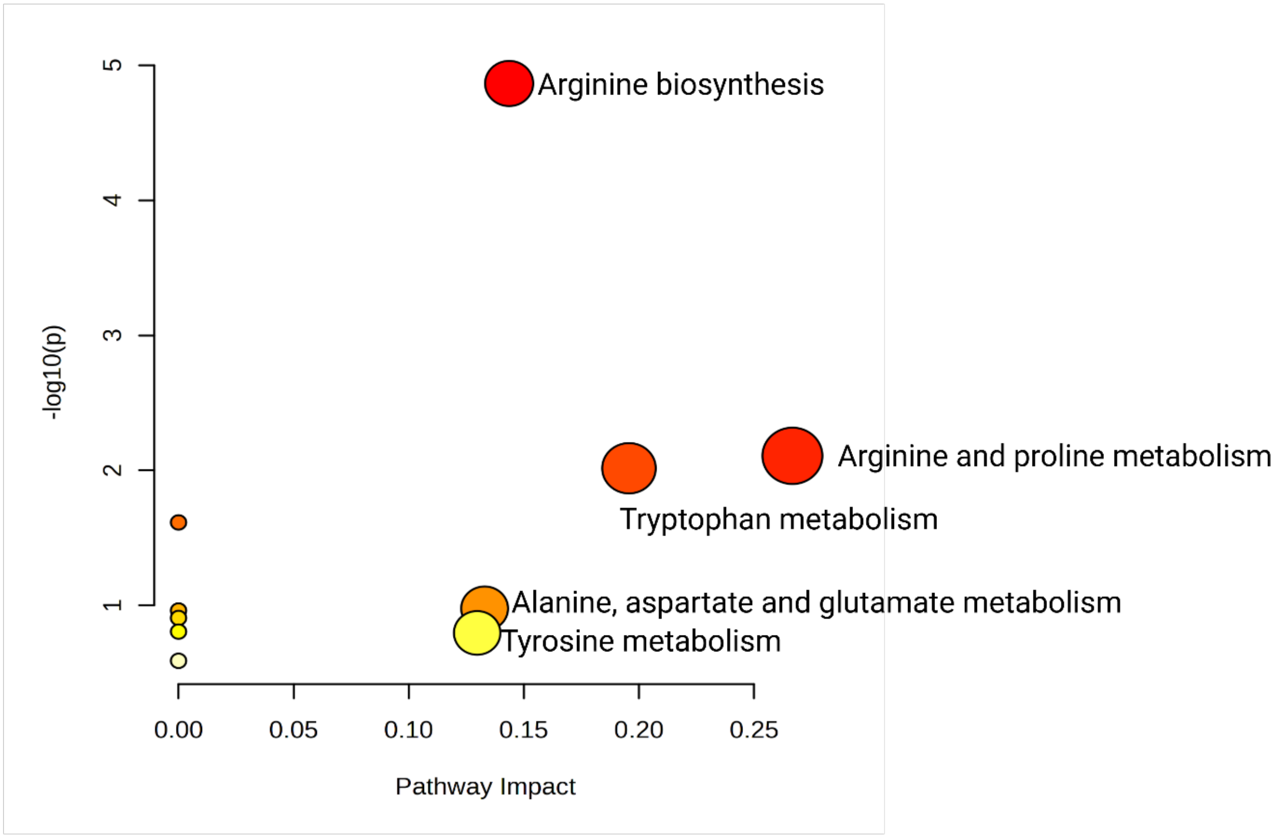

Figure S8. Predicted impacted metabolic pathways in larval zebrafish based on metabolic shifts. Significant metabolites were analyzed for enrichment in zebrafish-specific KEGG pathways using MetaboAnalyst 6.0 to contextualize observed shifts in metabolite abundances. Several pathways were identified, including arginine biosynthesis, arginine and proline metabolism, tryptophan metabolism, alanine, aspartate and glutamate metabolism, and tyrosine metabolism.

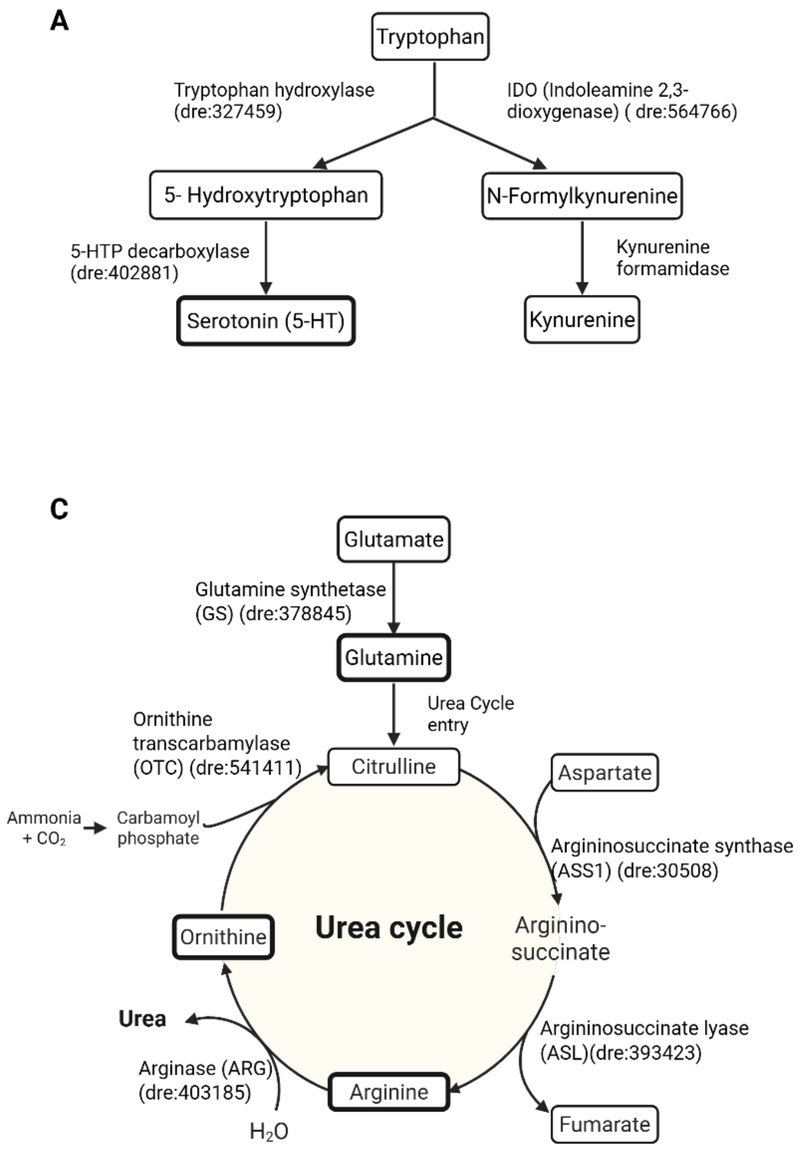

Figure S9. Visualization of predicted impacted metabolic pathways. Zebrafish-specific KEGG pathways illustrating key metabolic routes potentially impacted by colonization status and/or azoxystrobin exposure. Simplified pathways include (A) tryptophan metabolism, (B) tyrosine metabolism, and (C) an integration of arginine biosynthesis and metabolism, arginine and proline metabolism, and alanine, aspartate, and glutamate metabolism. Metabolites significantly affected by treatment are shown in bold (serotonin, dopamine, glutamine, arginine, and ornithine), with zebrafish-specific enzymes and genes identified and annotated in each pathway. Created in BioRender. Wray, C. (2026) https://BioRender.com/zb6zdcl
