# Supplementary material for: Integration of human microbiota (SIHUMIx) and zebrafish models reveals microbiome-mediated host responses to azoxystrobin
Source: Toxicol Sci. 2026 Mar 7;209(4):kfag022. doi: 10.1093/toxsci/kfag022 (PMC13105170; doi:10.1093/toxsci/kfag022)
Supplement: kfag022_Supplementary_Data [file kfag022_supplementary_data.zip › 03-Apr-2026_090434_20260203_supp_methods_AZO.pdf]

Supplemental Material

Integration of human microbiota (SIHUMIx) and zebrafish models reveals microbiome-mediated host responses to azoxystrobin

Chloe Wray[1,3*], Victor Castañeda-Monsalve[2*,5+], Beatrice Engelmann[2], Ulrike E. Rolle-Kampczyk[2], Nicole Schweiger[1], Sebastian Gutsfeld[1], Debjyoti Ghosh[2], Siraz Kader[1], Charles R. Tyler[3], Nico Jehmlich[2#], Tamara Tal[1,4#]

[1]Department of Ecotoxicology, Chemicals in the Environment Research Section, Helmholtz-Centre for Environmental Research – UFZ, Leipzig, Germany

[2]Department of Molecular Toxicology, Chemicals in the Environment Research Section, Helmholtz-Centre for Environmental Research – UFZ, Leipzig, Germany

[3]University of Exeter, Biosciences, Geoffrey Pope Building, Stocker Road, Exeter, Devon EX4 4QD, UK

[4]Medical Faculty, University Leipzig, Leipzig, Germany

[5]Proteomics Research Infrastructure, Faculty of Health and Medical Sciences, University of Copenhagen, Denmark

[*]Indicates equal contribution

[+]Indicates current affiliation

[#]Corresponding Authors: Helmholtz Center for Environmental Research – UFZ, Permoserstrasse 15, 04318 Leipzig, Germany. Email: nico.jehmlich@ufz.de; **tamara.tal@ufz.de**

Supplemental Methods

Section 2.1 Supp.: Simplified Human Microbiota Model (SIHUMIx)

This microbiota model was originally developed in murine models using bacterial isolates from the human gastrointestinal tract. It reflects the dominant phyla and major metabolic functions of the human gut microbiota. Some strengths of the model are that it maintains functional capacities in murine systems that are comparable to those observed in hosts with native microbiota, can be effectively adapted to *in vitro* environments, and is composed of species that are thoroughly characterized both genetically and proteomically.

Section 2.3 Supp.: Metaproteomic analysis sample preparation

Single-Pot Solid-Phase-enhanced Sample Preparation (SP3)

After the protein extraction, a 30 µg amount of protein amount was transferred into to a 1.5 mL Eppendorf tube (Hamburg, Germany), and the volume of the sample was brought to 200 µL using 100 mM Tetraethylammonium bromide buffer (TEAB). The proteins were then subjected to a reduction step using 2.5 mM tris(2-carboxyethyl) phosphine hydro-chloride (TCEP) for 1 at 55°C), followed by alkylation with 9 mM iodoacetamide (IAA) for 30 minutes at room temperature in the dark. Following alkylation, 260 µL of acetonitrile (ACN) was added to each sample.

To facilitate protein binding, 50 µg of Sera-Mag Carboxylate-Modified Magnetic Beads and SpeedBeads (10 mg/mL, Cytiva, Marlborough, Massachusetts) were added. Samples were incubated at room temperature for 8 minutes, then placed on a magnetic rack for 2 minutes to allow bead separation. The supernatant was removed, and the beads were washed twice with 200 µL of 70% ethanol, followed by a single rinse with 200 µL ACN. After each wash, the beads were allowed to rest on the magnetic rack from 2 minutes before supernatant removal.

Once air-dried, beads were resuspended in 5 µL of digestion buffer (100 mM TEAB containing 0.6 µg of Trypsin/Lys-C Mix, Mass Spec Grade; Promega, Madison, United States) and incubated at 37°C for 16 hours for the enzymatic digestion. After digestion, 150 µL of ACN was added to reach a final concentration of above 95% ACN. Samples were incubated at room temperature for 8 minutes, then placed on a magnetic rack for 2 minutes and the supernatant was discarded. A final rinse was carried out with 200 µL of 100% ACN, followed by magnetic separation on the magnetic rack and removal of residual liquid.

The bound peptides were eluted by reconstituting the beads in 50 µL of 2% DMSO, sonicating for 1 minute, and allowing 2 minutes for incubation on the magnetic rack for peptide recovery. A second elution under the same conditions was conducted excluding sonication, yielding a total peptide volume of 100 µL. These eluates were vacuum-dried and reconstituted in 30 µL of 0.1% formic acid (FA). After centrifugation at 20,000 x g for 10 minutes to remove residual beads, 25 µL of the clarified peptide solution was transferred to LC-MS vials and stored at -80°C until spectrometric analyses.

Section 2.4 Supp.: Metaproteomics - LC-MS/MS measurements

For each nanoLC-MS run, 1 µg of peptides was injected into a Vanquish Neo nanoHPLC system (Thermo Fisher Scientific). Peptide trapping was performed using a C18 reverse-phase precolumn (Acclaim PepMap™ 100, 75 µm × 2 cm, 3 µm particle size, nanoViper; Thermo Fisher Scientific), followed by separation on an analytical C18 column (PepMap™ Neo, 75 µm × 150 mm, 2 µm particle size, Double nanoViper; Thermo Fisher Scientific).

Chromatographic separation was achieved using a two-step linear gradient with mobile phase A (0.01% formic acid in water) and B (80% ACN in water containing 0.01% formic acid). Over the initial 65 minutes, the concentration of mobile phase B was gradually increased from 4% to 30%, followed by a second gradient phase

lasting 30 minutes during which it increased from 30% to 55%. A constant flow rate of 300 nL/minute was maintained throughout the separation.

Peptides were ionized via the Nanospray Flex™ Ion Source (Thermo Fisher Scientific) and analyzed using an Orbitrap Exploris™ 480 mass spectrometer (Thermo Fisher Scientific) operated in data-independent acquisition (DIA) mode. MS1 scans were acquired over an *m/z* range of 350-1,500 with a resolution of 120,000, an AGC target of 3,000,000, and automatic maximum injection time. MS/MS acquisition was conducted at a resolution of 30,000, using an AGC target of at 1,000,000, normalized collision energy of 30%, RF lens setting of 45%, using an auto injection time. DIA was performed using a windowing scheme, with an isolation width of 24 *m/z* and a1 *m/z* overlap across the precursor range of 350-1,500 *m/z*.

Section 2.6 Supp.: Metaproteomic DIA data processing

Protein identification relied on the same species-specific protein sequence databases used for the spectral library generation. Retention time prediction was facilitated by the indexed Retention Time (iRT) method. Functional annotation of identified proteins was performed by submitting their sequences to the Kyoto Encyclopedia of Genes and Genomes (KEGG) via the GhostKoala tool, which assigns KEGG orthologs. Label-free quantification was used for relative abundance of each SIHUMIx species. This involved summing the intensities of all proteins mapped to each microorganism and expressing them as a fraction of the total bacterial protein intensity within each sample. For downstream functional analyses, only KEGG pathways represented by at least five proteins and a minimum of 15% coverage were considered.

Section 2.7 Supp.: Short Chain Fatty Acids (SCFA) analysis for SIHUMIx

To prepare samples for derivatization, ACN was added to adjust the final concentration to 50%. For derivatization, an equal volume of two reagents was added: 200 mM 3-nitrophenylhydrazine and 120 mM N-(3-dimethylaminopropyl)-N-ethylcarbodiimide hydrochloride, both prepared in pyridine. The mixture was incubated at 40 °C for 30 minutes with shaking at 300 rpm. Following derivatization, the SCFA-containing solutions were diluted 1:50 using 10% ACN.

An aliquot of 10 µL from the diluted, derivatized solution was injected into an RSLC UltiMate 3000® system (Thermo Fisher Scientific), interfaced with a QTRAP 5500® mass spectrometer (AB Sciex, Framingham, MA, USA). Chromatographic separation of SCFAs was performed using an Acquity UPLC BEH C18 column (1.7 µm particle size; Waters, Eschborn, Germany), employing a binary solvent system: water with 0.01% formic acid (solvent A) and ACN with 0.01% formic acid (solvent B). The column temperature was maintained at 40 °C, with a constant flow rate of 0.35 mL/min.

The elution gradient consisted of an initial hold at 15% B for 2 minutes, followed by a gradient from 15% to 50% B over a period of 15 minutes. This was followed by 1 minute at 100% B and a 3-minute re-equilibration at 15% B. SCFAs were detected and quantified using a scheduled multiple reaction monitoring (MRM) method with compound-specific transitions for each SCFA. Chromatographic peak areas were processed using Analyst® Software (v1.6.2, AB Sciex), and the resulting data were exported for downstream analysis. Quantification of metabolites was carried out using custom R scripts (R Studio v2023.03.0) and calibration curves.

Section 2.8 Supp.: Untargeted metabolomics

Before undergoing analysis, samples were combined with five volumes of solvent mixture containing MeOH: ACN:H_20 in a 2:3:1 (v:v:v) ratio. The mixtures were sonicated and subsequently centrifuged before 550 µL of the resulting supernatant was transferred and evaporated. For LC-MS measurements, dried samples were reconstituted in 100 µL 0f 0.1% formic acid and 1% ACN in water. A pooled sample was prepared by equal volumes from each resuspended sample and was subsequently spiked with a mix of internal standards to a final concentration of 5.5 µM.

Metabolite profiling was performed on a high-performance liquid chromatography (HPLC) system (Vanquish, Thermo fisher Scientific, MA, USA) coupled to a mass spectrometer (Orbitrap IQ-X, Thermo Fisher Scientific, MA, USA), in AcquireX mode (Thermo Fisher Scientific, MA, USA). Each sample was analyzed in duplicate across two separate runs run in two separate batches, the first batch in positive ionization mode and the second in negative.

For each injection, 5 µL of the sample was loaded onto an HPLC system and initially trapped on a precolumn (ACQUITY UPLC HSS T3 VanGuard precolumn, 2,1mm X 5mm,186003976, Waters GmbH, Eschborn, Germany). The sample was subsequently separated on an analytical column (ACQUITY UPLC HSS T3 Columns,2.1 mm X 100 mm, 186003539, Waters GmbH, Eschborn, Germany) using a solvent flow rate of 0.3 mL/min and a column temperature of 40°C. Chromatographic separation was achieved using a gradient of solvent A (Milli-Q water with 0.1% formic acid) and solvent B (ACN with 0.1% formic acid).The run began with 99% solvent A and 1% solvent B for the initial 3 minutes. Solvent B was increased in a linear manner to 35% by 15 minutes, and increased further to 99% by 20 minutes. This composition was held for 2 minutes (until 22 minutes), followed by a rapid drop to 1% by 22.5 minutes. The gradient was held at 1% B until the conclusion of the 25-minute run.

Eluting analytes were ionized using an electrospray ionization (ESI) source (Ion-Max NG, Thermo fisher Scientific, MA, USA), operated in either in positive mode (3400 V) or negative mode (-2600 V). Source settings were configured as follows: sheath gas at 40 units, auxiliary gas at 5 units, sweep gas at 1-unit, vaporizing temperature at 400°C, and ion transfer tube temperature at 300°C.

Metabolite identification was carried out using a combination of MS/MS and Full-MS acquisition modes. First, a blank injection sample containing only solvent A was used to identify and exclude background contaminant features. To detect features, a pooled sample was then analyzed in Full-MS mode, followed by five successive MS/MS runs of the same pooled sample. In each MS/MS run, features previously fragmented were excluded from MS2 acquisition. This enabled the fragmentation of unique features and maximized coverage of metabolites identified in ensuing data analysis. Following this, all individual study samples were analyzed with the Full-MS method. To monitor instrument performance and ensure data quality, pooled quality control (QC) samples were injected at the beginning, midpoint, and end of each batch. All QC injections consisted of four pooled replicated analyzed using the Full-MS method to assess analytical reproducibility and stability across run.

For metabolite identification, MS/MS spectra were acquired with the following settings: The Orbitrap analyzer was used at a resolution of 60,000 MS1. Quadrupole isolation was applied, with a scan range of 50-1000 m/z and a maximum injection time 50 ms. The RF Lens voltage was set to 35%. Polarity was set according to the batch, with positive mode for positively ioned samples and negative mode for negative batches. A filtering threshold was applied to include only precursor ions exceeding an intensity of 20,000. Dynamic exclusion parameters were set to exclude a feature after a single detection, with an exclusion duration of 2.5 seconds, and a mass tolerance window of ±5 ppm. Isotopes were excluded, and an exclusion was applied within-cycle to maximize unique precursor selection.

For targeted MS2, a mass list (m/z) was generated using Xcalibur AcquireX (Thermo fisher Scientific, MA, USA), with a start/end time mode, an intensity threshold, and mass tolerance limits of ±5 ppm. MS2 acquisition was performed using the Orbitrap detector with quadrupole isolation and a 1.5 m/z isolation window. Higher-energy collisional dissociation (HCD) was used as the activation method with stepped, normalized collision energies set at 30%, 32%, and 35%. The Orbitrap resolution was set to 30,000, with scan range mode on auto. AGC target was set to standard, and the maximum injection time was configured as custom at 50 ms. One microscan was collected per scan, and the data were recorded in profile mode.

For relative quantification, samples were analyzed using a Full-MS method with the following settings: Orbitrap resolution was set to 120,000, with a scan range of 50-1000 m/z, a maximum injection time 50 ms, and an RF lens voltage of 35%. Polarity was set to match that of the acquisition batch.

All data were collected in *.RAW format and processed using Compound Discoverer^{TM} (Thermo fisher Scientific, MA, USA). The resulting output included normalized peak abundances and compound annotations. To ensure data quality, only peaks meeting the following criteria were retained: presence of an associated MS2 spectrum, a minimum mzCloud best match score of 50, a peak rating of ≥ 3.5 in at least two samples, and a mass deviation of less than 5 ppm from the theoretical mass of the compound. In cases where a compound was associated with multiple peaks at distinct retention times, only the peak exhibiting the highest normalized abundance across all samples was retained for analysis. Furthermore, any additional peaks assigned to the same compound and eluting within a 30-second window of the most abundant peak had their intensities summed with that of the primary peak.

Section 2.20 Supp.: 16 rRNA of whole zebrafish larvae and flask media

2.20a Sample preparation

Bacterial DNA was extracted from whole larvae using the FastDNATM SPIN Kit for Feces (MP Biomedicals, USA) with modifications. Since the samples were not dry, steps 1 and 2 of the manufacturer's protocol were omitted. The following modifications were made to steps 3 through 17: Pools of frozen larvae were transferred from the 1.5 mL tubes (Eppendorf Tubes®) to Lysing Matrix E tubes of FastDNATM SPIN Kit for Feces (MP Biomedicals, USA) using 489 µL of sodium phosphate (NaPO4) buffer. For each sample, an additional 61 µL of MT buffer was added, vortexed briefly, and homogenized using FastPrep 5G instrument (MP Biomedicals) at 6.5 m/s for 40 seconds. Homogenization was performed twice, with samples being cooled on ice for 2 min between cycles before being centrifuged at 13,000 rpm for 6 min. This process yielded approximately 600 µL of supernatant which was then transferred to a fresh 2 mL microcentrifuge tube. Each tube then received 125 µL of PPS solution followed by shaking to ensure thorough mixing before being incubated at 4°C for 10 min. After incubation, samples were centrifuged for 3 min at 13,000 rpm. During this three-minute period, 500 µL of Binding Matrix solution was added to a new set of 2 mL centrifuge tubes. The resultant supernatant of each sample was transferred to the tubes with Binding Matrix solution. Samples were mixed by pipetting up and down then transferred to a Thermo Mixer, where they were stirred for 5 min at 500 rpm. Next, the tubes were centrifuged for 3 min at 13,000 rpm, and the supernatant was gently decanted to avoid disrupting the pellets. Pellets in each tube were resuspended in 500 µL of Wash Buffer #1, then transferred to a spin filter tube for centrifugation at 13,00 rpm for 2 min. This process was repeated for the remaining suspension, with the flow-through discarded after each step. To resuspend the pellets again, 500 µL of Wash Buffer #2 was added to the spin filters before centrifugation at 13,000 rpm for 3 min, after which the flow-through was discarded. To remove any remaining ethanol from the binding matrices and dry the spin filter tubes, centrifugation was performed at 13,000 rpm for 2 min. In the last steps of the extraction, the spin filters were transferred to a new 2 mL microcentrifuge tube where 60 µL of TES buffer was added to resuspend the pellets. To elute the purified DNA, the tubes were centrifuged at 13,000 rpm for 3 min, yielding approximately 60 µL of eluted DNA per sample. To assess DNA quality (260/280 ratio) and quantify DNA concentration (ng/ µL), 1 µL of each sample was measured in triplicate using a Nanodrop spectrophotometer. For 16S rRNA sequencing of whole zebrafish larvae, samples were normalized to 20 ng/ µL using TES Buffer to yield 50 µL of the final sample. Remaining, non-normalized, DNA was archived at -20°C.

Section 2.21b Supp.: Statistics for zebrafish metabolomics

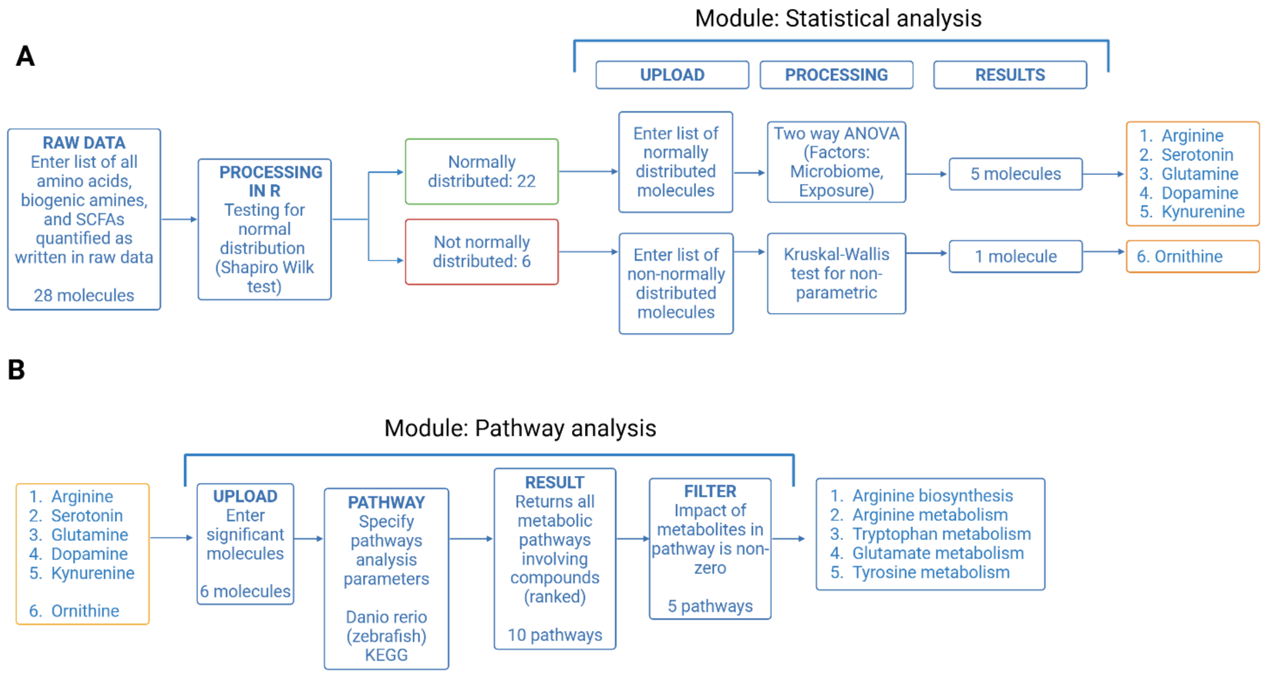

Schematic representing the data filtering for relevant metabolic pathways in larval zebrafish. (A) Initial data processing was performed in R in order to assess the distribution of each compound using the Shapiro-Wilk test. Compounds that were normally distributed were analyzed in MetaboAnalyst 6.0 using the Statistical Analysis [metadata table] module. Significant effects of azoxystrobin, colonization status, or their interaction were determined using the ANOVA2 (two-way ANOVA) feature. For compounds that were not normally distributed, each factor was analyzed separately using the Statistical Analysis [one factor] module with the Non-parametric ANOVA (Kruskal-Wallis) setting to identify significant effects ($p < 0.05$). (B) Significantly affected compounds identified in the previous step were processed in the Pathways Analysis module of MetaboAnalyst6.0 to determine which pathways may be affected.
